# Measures of Perceived Neighborhood Food Environments and Dietary Habits: A Systematic Review of Methods and Associations

**DOI:** 10.3390/nu14091788

**Published:** 2022-04-24

**Authors:** Miwa Yamaguchi, Panrawee Praditsorn, Sintha Dewi Purnamasari, Kitti Sranacharoenpong, Yusuke Arai, Samantha M. Sundermeir, Joel Gittelsohn, Hamam Hadi, Nobuo Nishi

**Affiliations:** 1 International Center for Nutrition and Information, National Institutes of Biomedical Innovation, Health and Nutrition, 1-23-1 Toyama, Shinjuku-ku, Tokyo 162-8636, Japan; nnishi@nibiohn.go.jp; 2 Institute of Nutrition, Mahidol University, 999 Phuttamonthon 4, Salaya, Phuttamonthon, Nakhon Pathom 73170, Thailand; panrawee.pra@mahidol.ac.th (P.P.); kitti.sra@mahidol.ac.th (K.S.); 3 Alma Ata Graduate School of Public Health, University of Alma Ata, Jl. Brawijaya 99, Tamantirto, Yogyakarta 55183, Indonesia; sinthadewips@almaata.ac.id (S.D.P.); hhadi@almaata.ac.id (H.H.); 4 Department of Nutrition, Chiba Prefectural University of Health Sciences, 2-10-1 Wakaba, Mihama-ku, Chiba-shi 261-0014, Japan; yusuke.arai@cpuhs.ac.jp; 5 Center for Human Nutrition, Department of International Health, Bloomberg School of Public Health, The Johns Hopkins University, 615 North Wolfe St, Baltimore, MD 21205, USA; srex2@jhmi.edu (S.M.S.); jgittel1@jhu.edu (J.G.)

**Keywords:** food environments, perceived measurements, food access

## Abstract

Access to healthy food is a necessity for all people. However, there is still a lack of reviews on the assessment of respondent-based measures of neighborhood food environments (perceived food environments). The aim of this systematic review was to evaluate the measurement tools for perceived food environments by five dimensions of food access and to obtain the overview of their associations with dietary habits among people aged 18 years and older in middle- and high-income countries. Observational studies using perceived food environment measures were identified through a systematic review based on two databases for original studies published from 2010 to 2020. A total of 19 final studies were extracted from totally 2926 studies. Pertaining to the five dimensions of food access, 12 studies dealt with *accessibility*, 13 with *availability*, 6 with *affordability*, 10 with *acceptability*, 2 with *accommodation*, and 8 with a combination of two or more dimensions. Perceived healthy food environments were positively associated with healthy dietary habits in 17 studies, but 8 of them indicated statistically insignificant associations. In conclusion, this review found *accessibility* and *availability* to be major dimensions of perceived food environments. The relationship between healthy food environments and healthy diets is presumably positive and weak.

## 1. Introduction

The United Nations sustainable development goals include Zero Hunger, a goal targeted at ending hunger, achieving food security, and improving nutrition [1]. Food environments are characterized by the availability, affordability, convenience, promotion, quality, and sustainability of foods and beverages in wild, cultivated, and built spaces [2]. Healthy food environments are essential to ensure food security, such that all citizens can sustainably access healthy food [3]. Empirical evidence of the health impact of neighborhood food environments has accumulated especially in deprived areas in high-income countries since the early 1990s [4]. A review [5] has reported inequalities in food access in the United States, and there has been a paucity of studies in other developed countries.

Proper measurement of food environments is required to investigate the relationship between food environments and dietary habits. Objective measures of food environments, such as geographic information systems, are direct observations and are a common methodology for assessing food environments [6]. However, these objective measures may not necessarily capture individual behaviors or the actual situation of food access [7]. For example, studies conducted in the United States have demonstrated that consumers traveled beyond their nearest supermarket to obtain cheaper [8] and healthier food [9], indicating that physical distance may not be the only factor involved in choosing primary food stores. Perceived (respondent-based) measures, such as individual perceptions and experiences may support the limitations of objective measures for assessing food environments.

Nevertheless, perceived measures face some challenges. First, there are no standardized measures of perceived food environments. One of the processes for developing a standardized measurement is to classify them based on the different aspects of food environments. Penchansky and Thomas [10] proposed the utilization of the five dimensions of “food access” (*accessibility*, *availability*, *affordability*, *acceptability*, and *accommodation*). Glanz et al. [11] suggested that community and consumer environments impact individual behaviors. Accordingly, *accessibility*, *availability*, and *accommodation* can be included in the community environment, and *affordability* and *acceptability* can be grouped in the consumer environment. Second, there is still a lack of evidence on the relationship between perceived food environments and dietary habits. Only one review [12] classified the perceived and objective measures, and indicated that perceived measures of *availability* within the neighborhood food environments were consistently associated with healthy diets among studies published through 2011. However, a review [12] targeted both children and adults, wherein it was reported that food environments for children may be influenced by the household main shopper. A review targeting adults who are likely to be the main shoppers is required.

The aims of this study were to systematically review existing tools used to measure perceived food environments according to the five dimensions of “food access”, and to assess the association of perceived food environment measures with dietary habits.

## 2. Materials and Methods

### 2.1. Search Strategy

**Figure 1:**
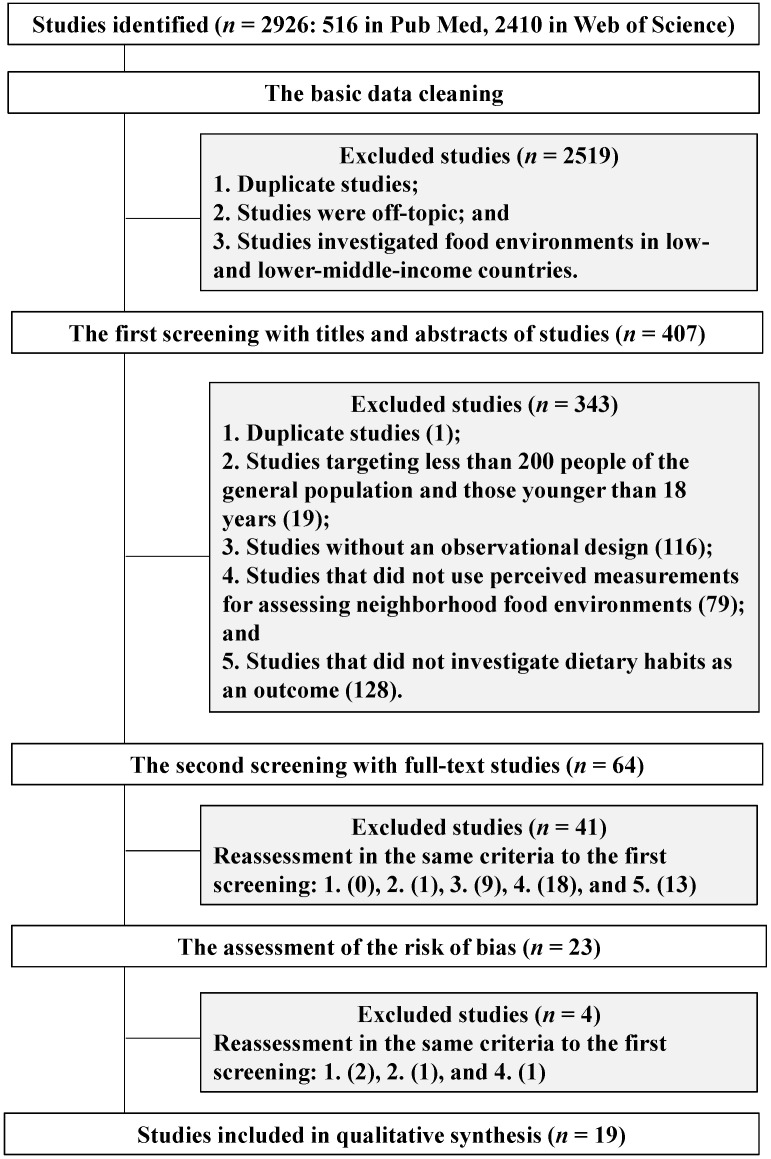
Flow chart of data extraction.

The review protocol was registered in the public domain (PROSPERO registration number: CRD42020201881) in accordance with the PRISMA guidelines [13]. The present systematic search was conducted to identify studies with observational designs published online in English, between 1 January 2010 and 6 August 2020, using the PubMed and Web of Science databases (Figure 1). Systematic keyword searches were developed and agreed upon by all authors to identify studies that investigated the relationship between perceived food environments and dietary habits among community-dwelling people aged 18 years and older in middle- and high-income countries, with at least 200 people [14] (Supplementary Materials Tables S1 and S2). The two databases were explored by one reviewer on 6 August 2020, to unify the run time, and duplicates were excluded. Basic data cleaning was performed before the first screening. The articles were screened to identify those that failed to be eliminated in the keyword search: off-topic studies and those that investigated food environments in low- and lower-middle-income countries [15]. We defined studies as off-topic when relevant text words, such as “food environments”, “dietary habits”, and “food access” were not included in the title or the abstract. In the first screening, we excluded studies that stated the following in the title and abstract: (1) duplication, (2) the criteria of the number of population, (3) studies without an observational design, (4) studies that did not use perceived measurements for assessing neighborhood food environments, and (5) studies that did not investigate dietary habits as an outcome. The second screening was conducted by investigating the full text using the same exclusion criteria as the first screening. The basic data cleaning and first- and second-screenings of studies were independently conducted, blinded, and then jointly reviewed by three reviewers using the free web application of Rayyan [16]. The three reviewers assessed the risk of bias individually, although not blinded, and decisions were confirmed by four other reviewers.

### 2.2. Analyses

We summarized the studies by the number of study participants, location (i.e., urban and rural), country, project data source, study design, and target population. Furthermore, we described the measurement tools and types of analyses (i.e., continuous and dichotomous) of perceived food environments and dietary habits, respectively. Measures of perceived food environments were classified according to the five dimensions of food access (*accessibility, availability, affordability, acceptability,* and *accommodation*) [10]. The definition of the five dimensions are as follows [10,12]: *accessibility*, the location of the food supply source and the ease of getting to that location, accounting for travel time and distance; *availability*, the adequacy of the supply of healthy food; *affordability*, food prices and people’s perceptions of worth relative to the cost, which is often measured by store audits of specific foods, or regional price indices; *acceptability*, people’s attitudes about attributes of their local food environment, and whether the given supply of products meets their personal standards; and *accommodation*, how well local food sources accept and adapt to the needs of local residents (e.g., store hours and types of payment accepted). Dietary outcomes were classified by measures of fruit and/or vegetable intake, other healthy food intake, unhealthy food intake (i.e., fast food), and a diet-quality index that the selected studies employed. We identified healthy and unhealthy foods according to the definitions of the selected studies.

The risk of bias was assessed across seven domains, each of which consisted of three to nine indicators based on two scales: the Risk of Bias for Nutrition Observational Studies Tool [17,18], which is applicable to observational studies in public health nutrition, and the Newcastle Ottawa Scale [19] which indicates potential biases of food environment studies (Supplementary Materials Table S3). The seven domains were (1) confounding, (2) selection of participants, (3) classification of exposures, (4) departures from intended exposures, (5) missing data, (6) measurement of outcomes, and (7) selection of reported results.

Finally, we summarized the associations of perceived food environments with dietary habits in the final model of the analysis. The association was defined as “positive” when healthy perceived food environments were significantly associated with a higher intake of healthy food or a lower intake of unhealthy food; and was defined as “negative” when healthy perceived food environments were significantly associated with a lower intake of healthy food or a higher intake of unhealthy food. To consider not only the statistical significance but also the trend of the association [20], we assessed the trend even if the association was statistically insignificant. We counted the studies on the use of the five dimensions of food access among the selected studies. We counted each dimension when two or more dimensions were reported in one study. In addition, we counted studies that indicated positive or negative associations in the indicator of the dimension. When two or more indicators were indicated in one dimension, we counted them as one statistically significant association. For example, we counted one significant association from six types of indicators in one dimension of *affordability* in one study [21]. Statistical significance was set at a two-sided *p*-value of 0.05.

We decided not to conduct a meta-analysis because of the heterogeneity in exposure and outcome measurements across the studies.

## 3. Results

### 3.1. Study Overview

Among the 2926 studies identified by the two databases, 2519 were excluded during the basic data cleaning (Figure 1). Of the remaining 407 studies, we excluded 343 that met the exclusion criteria in the first screening. After a full article review during the second screening, 41 of the 64 studies were excluded. Of the 23 studies remaining, we excluded an additional four studies at the risk of bias assessment. Of these four, one study [22] was excluded because it targeted a specific population that received healthcare services, and another [23] did not use a perceived measurement tool for food environments; two sets of studies used the same measurement tools from the same research project: Lucan et al. [24] and Lucan and Mitra [25]; Bivoltsis et al. [26] and Trapp et al. [27]. We selected Lucan and Mitra [25] and Bivoltsis et al. [26] targeting a wider study areas and larger population.

Among the indicators in the 19 studies, we omitted the indicator of home food environment in the studies of Alber et al. [28], Kegler et al. [29], and Springvloet et al. [30], because the current review did not focus on household food environments. However, we did not omit the indicator of the *accessibility* of unhealthy food at the workplace, as investigated by Carbonneau et al. [31], because the indicators of neighborhood and workplace were integrated into one score.

### 3.2. The Assessment of the Risk of Bias

There was no serious or critical risk of bias in the 19 studies, and most of them had a moderate or low risk of bias against the seven classified domains (Supplementary Materials Table S4). All studies were determined to have a moderate risk of bias in confounding, selection of participants, and departure from intended exposures. The moderate risk of bias against the classification of exposures (i.e., perceived food environments) was identified in three studies; one study [32] did not mention the validity or reliability of the perceived measurement tools, and two studies [30,33] used the indicators of perceived food environments that were employed in previous reports but did not investigate validity and reliability. Four studies that were judged as having a low risk of bias of missing data performed multiple imputation [31,34], engaged in a listwise deletion of data by not observing a missing data pattern [35], and did not exclude missing data amounting to 1.5% of the total [25]. Five studies [7,28,29,36,37] did not describe missing data. Seven studies [7,25,35,38,39,40,41] with a low risk of bias in measurement outcomes (i.e., dietary habits) used data from interviews conducted by trained interviewers and not through self-description of participants.

### 3.3. Characteristics of the Study Design

Thirteen studies were conducted in the United States, and five studies [21,26,30,31,37] were conducted in Western countries and Australia in the Oceania region (Table 1). Only one study [33] has been conducted in Japan in East Asian countries. The study areas of 10 studies [7,21,26,28,30,31,35,37,38,42] were urban areas, while six studies [25,33,39,40,41] were conducted in both urban and rural areas. With respect to the study design, 18 studies were cross-sectional, and only one study [26] used a longitudinal study design from baseline 2003–2005 to 2004–2006 to investigate changes in food environments and dietary habits. Two studies included minority populations, such as French-speaking adults [31] and African American (with White) adults [29]. Three studies [7,35,42] targeted adults with low income. Furthermore, Lo et al. [34] targeted middle-aged and older women, and Sharkey et al. [32], and Yamaguchi et al. [33] targeted older adults.

### 3.4. Overview of the Measurement Tools of Perceived Food Environments

The frequency of usage of dimensions of food access were 12 studies in *accessibility*, 13 studies in *availability*, six studies in *affordability*, 10 studies in *acceptability*, and 2 studies in *accommodation* (Table 2). Studies have integrated two [29,35,36,39], three [32,34,37], and five [31] dimensions to form one score. Chapman et al. [21] used one dimension of *affordability* with three indicators, and five studies [7,26,33,41,42] used one dimension with a single-item indicator; three studies [7,26,33] used *accessibility*, one study used *availability* [41], and the other used *acceptability* [42].

A total of 17 studies used measurements of perceived food environments that were previously validated or pilot tested. Two studies [30,33] used measurements that were previously used but were not validated, and one study [32] did not validate the measurement. Indices of perceived food environments in five studies [25,28,35,38,40] exhibited moderate validity using objective measurements as a standard. Eight studies indicated that perceived measurements demonstrated a moderate level of internal consistency, as analyzed by test–retest reliability [7,28,29,34,38,39,41] and inter-item reliability [37].

Among the studies that used *accessibility*, there were four types of indicators for assessing neighborhood food stores: (1) the ease of access/or purchase of fruits and vegetables/or variety foods in the neighborhood [25,28,29,31,34,37,40]; (2) adequate quantities of neighborhood stores [33,36,37,39]; (3) walkable distance to the primary food stores [7,25,31]; and (4) convenient time (i.e., 10 to 15 min) to reach primary stores [26,31].

Eight studies [25,29,34,35,37,38,39,40] commonly referenced indicators of *availability* and/or *acceptability* proposed by Moore et al. [43,44,45], Mujahid et al. [46], Echeverria et al. [47], and/or Ma et al. [48]. Specifically, the dimension of *availability*—whether a large selection of fruits and vegetables was available in the neighborhood food environments—was investigated in six studies [29,34,35,37,38,39]. Ma et al. [40] investigated *availability* of healthy foods. *Acceptability*, which is the quality of fruits and vegetables, was investigated in six studies [25,34,35,37,38,39]. Other studies used different indicators but investigated *availability* of various healthy foods including vegetables [30,31,32], and *acceptability* of the quality of fruits and vegetables [28,31,32,42].

*Affordability* investigated the perception of worth relative to the cost of all food items [32] or the specific foods, such as fruits and vegetables [21,28,30,32,38], and healthy foods [31].

Indicators for assessing the influence of media on food and nutrition on one’s diet [31] and neighborhood social cohesion [29] were investigated by *accommodation*. Five studies investigated unhealthy food environments: *availability* of fast-food restaurants [41], *accessibility* of cafés or restaurants [26], and *accessibility* of fast-food restaurants in the score [31,36,37].

### 3.5. The Outcomes of the Dietary Habits

The intake of fruits and vegetables or only vegetables [30] was the most common method of measuring dietary habits. Two studies [25,41] investigated the frequency (times/week) of fast-food intake, and one study [29] investigated fat intake (Table 3). The intake of fruits and vegetables (servings, times, and grams per day) was calculated using the validated food frequency questionnaires [7,29,30,34,38,39,42] and the measurement tools that were previously used [25,33,40]. Score indices of diet quality were employed in four studies [26,31,36,37]. Bivoltsis et al. [26] used an unhealthy dietary score.

### 3.6. Overview of the Associations

Nine studies indicated significant positive associations of perceived food environments with healthy dietary habits within the dimensions of *accessibility* [7,33], *affordability* [21], *acceptability* [28,42], and mixed scores of *accessibility*, *availability*, *acceptability*, and/or *affordability* [32,34,36,37,39] (Table 4 and Table 5). Eight studies reported positive, but not statistically significant, associations [25,26,29,31,34,37,38,39]. Four studies showed significant negative associations of *availability* [28,30], *affordability* [30], and the mixed score of *availability* and *acceptability* [35] with healthy diets. Five studies [25,26,28,41] investigated the association of perceived food environments with unhealthy diets. Bivoltsis et al. [26] indicated a significant positive association of improved *accessibility* of healthy food environments with a high intake of unhealthy food. Bivoltsis et al. [26] also found that changing low *accessibility* to unhealthy food environments was significantly and positively associated with high intake of unhealthy food. A study conducted by Lucan et al. [25] showed that poor *accessibility* of fruits and vegetables and supermarkets and poor *acceptability* of grocery quality were significantly and positively associated with higher fast-food intake.

With respect to the statistical methods, 15 studies [7,21,25,26,28,30,31,32,33,34,36,37,38,41,42] used multivariate analyses to investigate the association adjusting for potential confounders, such as age, sex, ethnicity, income, and/or other social determinants of health. Using path analysis, four studies [29,35,39,40] investigated the pathways and mediations of perceived food environments in relation to dietary habits. Three studies [29,39,40] did not control for any possible confounders in the path model to prevent over-specification of the results [90]. Springvloet et al. [30] analyzed perceived food environments based on *availability* and *affordability* as mediators of the association between education level and vegetable intake using a linear regression model. Bivoltsis et al. [26] investigated the association of the change (improved and worsened) in perceived food environments with changes in the dietary habits of people after one to two years of changing residence in a longitudinal study.

## 4. Discussion

This is the first systematic review to assess the measures of the perceived food environments and their associations with dietary behaviors in middle- and high-income countries in 19 studies. *Accessibility* and *availability* were the most commonly measured dimensions of food access. A positive relationship between healthy perceived food environments and healthy dietary habits was observed among 17 studies, with nine of studies having a statistically significant relationship.

### 4.1. Characteristics of the Study Design

The reviewed studies mostly investigated food environments in the United States and other Western countries. Global changes in the food system associated with global economic growth have increased *availability* of unhealthy food [91] and consequently transformed dietary habits. Therefore, more evidence from different regions of non-Western countries, such as Asian countries, is required. No significant difference in the association of subjective food environments and dietary intake between urban and rural areas was observed in this review. However, studies in rural areas [29,32,34,36] considered the physical distance and/or number of healthy food stores in neighborhoods. This is because rural–urban inequality, such as infrastructure challenges and low population density, was in existence [92]. Therefore, specific strategies for rural communities are required.

In accordance with the present results, significant associations were observed in studies that targeted specific populations. For example, studies that targeted socially vulnerable people, such as those with low incomes [7,35,42] and older adults [32,33], indicated a significant association between perceived food environments and dietary habits. One review [93] proposed the stigma and food inequity conceptual framework which is composed of the structural (e.g., neighborhood infrastructure and targeted marketing) and individual (e.g., awareness and endorsement of negative beliefs, thoughts, and beliefs) levels. These stigmas are associated with food inequities due to access to resources, home food environments, and psychosocial and behavioral processes, which ultimately undermine healthy dietary intake and contribute to food insecurity [93]. To understand the food environments among vulnerable people, it is necessary to consider the contexts of poverty, race, nationality, gender, age, malnutrition, and their intersection.

### 4.2. The Assessment of the Risk of Bias

The moderate level of bias in confounding, selection of participants, intended exposures, and selection of results may be reasonable in the present review because the articles were observational studies that had limitations in the relevant confounder adjustment, eligible participant selection, and precise exposure setting compared to a well-designed randomized trial. We did not investigate the statistical power as heterogeneity in the exposure measurement and outcomes made comparing the effect size difficult, although we selected studies that targeted at least 200 people. A review observed that evidence depended on not only the statistical power but also the research methodology [94]. Therefore, this review assessed the risk of bias (i.e., study quality) comprehensively.

Regarding the bias of missing data, statistical approaches are expected to be considered for missing data in accordance with the missing patterns [95]. Four studies [25,31,34,35] considered proper imputation approaches in accordance with data missing completely at random, missing at random, and not missing at random [95]. A description of the statistical approaches for missing data is important to assess measurement bias. With respect to bias in dietary measurements, there were seven studies [7,25,35,38,39,40,41] with a low risk of bias as interviews were conducted by trained staff, which would reduce measurement error.

In the statistical model, all studies in this review considered confounders, such as age, sex, ethnicity, income, and/or other social determinants of health. However, only Bivoltsis et al. [26] considered the duration of residence in an area after relocation. The year of residence can possibly affect the geographic knowledge of the location of neighborhood stores and also impact the cultural *acceptability* of foods for people moving to a new neighborhood. Therefore, the duration of residence should be considered in food environment research.

### 4.3. Overview of Measurement Tools of Perceived Food Environments

Five studies [25,28,35,38,40] examined the validity of perceived food environments using objective measures as a standard. However, it is unclear whether objective (i.e., geographic) measurements accurately reflect the location of neighborhood primary food stores [8,9]. In addition, objective measurements are yet to be standardized using a consistent measure [12]. Nevertheless, using both objective and perceived measures is necessary to capture the complexity of food environments using different measurement tools.

According to the present review, *accessibility* of food stores within a walkable distance or convenient time, *availability* of a variety of fresh fruits and vegetables in the neighborhood could be some of the basic indicators to measure perceived food environments. In addition, *affordability* of prices of fruits and vegetables and *acceptability* of the quality of fruits and vegetables are necessary to consider the gap between individual perceptions and neighborhood retail. The indicators using *accessibility*, *availability*, *affordability*, and *acceptability* proposed by previous studies [43,44,45,46,47,48] could be optimized for structuring a food access measure, given that these indicators were employed in the present eight studies. These dimensions are useful and helpful from the viewpoint of public health to understand the measurement of perceived food environments the studies used.

However, the definition of dimensions have to be clearer since one dimension could overlap with another dimension. For example, certain studies were found to name only one dimension even when other dimensions were involved [29,31,35,37]. Most studies in this review did not clearly specify the dimensions. Especially in the definition of accommodation, convenience of store hours and types of payments are likely to be classified as *accessibility*, *availability*, and *acceptability*. The difficulty in the classification may limit the utility of these dimensions.

### 4.4. Overview of the Association of Perceived Food Environments with Dietary Habits

According to the present review, healthy perceived food environments are positively associated with healthy dietary habits but the association is weak. One study indicated that the individual-level factors accounted for the largest variation in fruit and vegetable intake as compared to that at the area level [96]. Nevertheless, health behaviors interact with physical and social environments, including food environments [97]. Therefore, interventions for both individual dietary behaviors and food environments may be important.

From the present results of the inverse relationship between healthy perceived food environments and unhealthy dietary habits, it is possible that people, especially those with low incomes [35], do not necessarily make healthy choices when both healthy and unhealthy foods are accessible, available, acceptable, and affordable. Indeed, the present review observed a significantly higher fast-food intake in healthy perceived food environments despite good *accessibility* of supermarkets/greengrocers [26]. Lucan and Mitra [25] indicated that poor *accessibility* of supermarkets, poor *availability* of produce, and poor *acceptability* of grocery quality were significantly associated with high intake of fast food. The present results imply that even when food environments are subjectively perceived as healthy, they may still have several choices of unhealthy food, given high *availability* of both healthy and unhealthy foods sold in stores according to consumer demands. A systematic review [98] investigated serving size labeling which is important to ensure an accurate understanding of nutritional content and food choices and consumption, among 14 articles (12 articles were from North America) and indicated that consumers in several studies had a poor understanding of serving size labeling. Another possibility would be suggested for why some people do not necessarily make healthy choices in good food environments. People who live in areas that are not familiar with healthy food may experience certain barriers in making healthy choices. One study reported that people who were introduced to the Mediterranean diet, which is considered a healthy diet, found a difficulty in purchasing food items due to an increase in food costs and found work, stress, and time pressures undermined adherence to the diet [99]. Therefore, nutrition education, as well as the improvement of food marketing, are required at the policy level to combat unhealthy food consumption so that people can effectively make healthy choices in complex food environments [100].

To determine food environments, a comprehensive assessment using both objective and subjective measures at structural and individual levels is required [93]. To terminate food inequity, it is necessary that policymakers collaborate with communities and private companies to devise a healthy city plan that includes measures, such as zoning the location of grocery/convenience stores. Simultaneously, further studies on the perceived food environments and literacy of healthy diets are required to monitor and assess the policy intervention.

### 4.5. Limitations

The study selection was conducted by three reviewers independently in the first and second screenings of the study records, keeping each decision blinded. However, the present review had some limitations that warrant mention. First, the present classification of perceived food environments according to the five dimensions of food access was inconclusive. The classification of the dimensions of food access on perceived food environments decided by the reviewers may differ from those of other reviewers. Second, there was a possibility of publication bias in the present review [101]. However, publication bias could be minimized by conducting a systematic review [13]; as a result, we extracted representative articles demonstrating evidence-based measurements and outcomes. Third, selection bias was not completely excluded, although it was minimized by blinding. Fourth, the causality of the relationship between perceived food environments and dietary habits is still unclear because all studies in this review were cross-sectional, except for one study [26]. Additionally, this review did not conduct the meta-analysis. A meta-analysis using longitudinal studies is needed to capture the causal relationship between perceived food environments and dietary habits, as perceived food environments change over time due to the turnover of food stores, constructed roads, and individual situations.

## 5. Conclusions

The order of frequent use of the perceived food environments was *availability*, *accessibility*, *acceptability*, *affordability*, and *accommodation*. Positive association of perceived food environments with dietary habits was observed, although this association may be weak. The characteristics of the relationship between perceived food environments and dietary habits are complex due to socioeconomic and latent background characteristics at the individual and community levels. Therefore, it is necessary to measure multiple aspects, such as the combination of food access dimensions of perceived food environments and to consider the effect of both healthy and unhealthy food in food environments and dietary habits.

## Figures and Tables

**Table 1:** Study designs of the reviewed studies.

Author, Year	*N*	Country	Location	Urban/Rural	Data Source	Study Design	Population
Alber et al., 2018 [28]	221	United States	Philadelphia (four neighborhoods)	Urban	Self-administered surveys between November 2010 and November 2011	Cross-sectional	Adults aged 18–65 years (average age 45.1 years)
Bivoltsis et al., 2020 [26]	1200	Australia	Perth	Urban	RESIDential Environments Project from 2003 to 2007	Longitudinal	Adults aged 18 years and older who plan to move into the new house by December 2005 (average age 40.5 years)
Carbonneau et al., 2019 [31]	1035	Canada	Québec	Urban	PRÉDicteurs Individuels, Sociaux et Environnementaux study from 2015 to 2017	Cross-sectional	French-speaking adults aged 18–65 years (18–34 years 36.6%)
Caspi et al., 2012 [7]	743	United States	Boston (Chelsea, Cambridge, and Someville)	Urban	The Health in Common study from February 2007 to June 2009	Cross-sectional	Adult residents aged 18 years and older (30–39 years 27.1%) resided in low-income housing
Chapman et al., 2017 [21]	2474	Australia	New South Wales	Urban	Part of a larger Community Survey on Cancer Prevention from January to February in 2013	Cross-sectional	Adults aged 18 years and older (median age 45.0 years)
Flint et al., 2013 [38]	1263	United States	Philadelphia (two low-income areas)	Urban	Philadelphia Neighbourhood Food Environment Study in the 2006 pre-intervention baseline	Cross-sectional	Primary adult shoppers aged 18 years and older in a household (average age 48.0 years)
Freedman et al., 2019 [35]	487	United States	Ohio (Cleveland and Columbus)	Urban	Baseline data from longitudinal quasi-experimental natural experiment from August 2015 to July 2016	Cross-sectional	Adults aged 18 years and older (average age 49.3 years) resided in low-income communities
Gase et al., 2016 [42]	1440	United States	Los Angeles (at public health centers)	Urban	The Los Angeles County Health and Nutrition Examination Survey II from February to April 2012	Cross-sectional	Adults aged 18 years and older (average age 55.0 years) with low income
Jilcott Pitts et al., 2015 [36]	366	United States	Eastern North Carolina	Rural	Baseline of Heart Healthy Lenoir Project from September 2011 to July 2012	Cross-sectional	Adults aged 18 years and older (average age 55.0 years)
Kegler et al., 2014 [29]	513	United States	Southwest Georgia	Rural	Baseline of Healthy Rural Communities 2 from September 2006 to March 2007	Cross-sectional	African American and White adults aged 40–70 years (average age 51.2 years)
Liese et al., 2014 [39]	831	United States	South Carolina (eight county regions)	Urban and rural	Telephone survey from April to July 2010	Cross-sectional	Adult shoppers aged over 18 years (average age 57.0 years)
Lo et al., 2019 [34]	513	United States	22 states	Rural	Baseline of StrongWomen Follow-Up Study in 2013	Cross-sectional	Midlife and older women (average age 67.0 years)
Lucan and Mitra, 2012 [25]	10,450	United States	Southeastern Pennsylvania (five countries, 991 census tracts)	Urban and rural	Public Health Management Corporation’s biennial random-digit–dialed Southeastern Pennsylvania Household Health survey from June to September in 2004	Cross-sectional	Adults aged 18 years and older (median age 47.0 years)
Ma et al., 2018 [40]	819	United States	South Carolina (eight counties)	Urban and rural	Telephone survey from April to July in 2010	Cross-sectional	Adults aged 18 years and older (average age 57.0 years)
Minaker et al., 2013 [37]	1170	Canada	Waterloo and Ontario	Urban	The Neighbourhood Environments in Waterloo Region: Patterns of Transportation and Health project from May 2009 to May 2010	Cross-sectional	Adults aged 19 years and older (average age 45.0 years in women and 44.7 years in men)
Oexle et al., 2015 [41]	838	United States	Central South Carolina (eight counties)	Urban and rural	Telephone survey from April to June in 2010	Cross-sectional	Adults aged 18 years old and older (average age 57.6 years)
Sharkey et al., 2010 [32]	582	United States	Texas and rural Brazos Valley Counties (six counties)	Rural	2006 Brazos Valley Health Assessment, the 2006–2007 Brazos Valley Food Environment Project, and the decennial 2000 U.S. Census Summary File 3	Cross-sectional	Older adults aged 60–90 years (average age 69.9 years)
Springvloet et al., 2014 [30]	1342	Netherlands	Five cities (Heerlen, Roermond, Venlo, Venray and Weert) in South of the Netherlands	Urban	Baseline data from a randomized controlled trial from March to October in 2012	Cross-sectional	Adults aged 20–65 years (average age 49.0 years)
Yamaguchi et al., 2019 [33]	83,384	Japan	31 municipalities in 12 prefectures	Urban and rural	The Japan Gerontological Evaluation Study in 2010–2011 survey	Cross-sectional	Older adults aged 65 years and older (average age 73.9 years)

**Table 2:** The measurement tools for perceived food environments.

Author, Year	Perceived Food Environments ^a^	Measurements	Variable Type
Alber et al., 2018 [28]	*Accessibility* (d) *Availability* (b) *Affordability* (c) *Acceptability* (a)	The measurement of the Nutrition Environment Measures Survey–Perceived [49] Perceived store consumer nutrition environment (a) Quality in neighborhood: Quality of fruits and vegetables in neighborhood (b) Availability in neighborhood: Availability of fruits and vegetables in neighborhood (c) Price in neighborhood: Price of fruits and vegetables in neighborhood (d) Ease of purchasing in neighborhood: Ease of purchase of fruits and vegetables in neighborhood Response: (a, b, d) 5-point agree/disagree Likert scale, (c) 4-point Likert scale (the higher score reflects healthy food environments)	Continuous
Bivoltsis et al., 2020 [26]	*Accessibility*	The Neighbourhood Environment and Walking Scale questionnaire [50] How long would it take to get from your home to the nearest cafe or restaurant/greengrocer/supermarket/if you walked to them? Response: within a 15 min walk of home or less; Yes (unhealthy food environments in café or restaurant and healthy food environments in greengrocer/supermarket) vs. No.	Dichotomous
Carbonneau et al., 2019 [31]	*Accessibility* (e, g, h, i, j) *Availability* (a, b) *Affordability* (d) *Acceptability* (c) *Accommodation* (f)	Perceived Food Environment Questionnaire [51] Six items of accessibility to healthy foods (a) I consider that the quantity of healthy foods offered by my main food retailer is sufficient (b) I consider that the variety of healthy foods offered by my main food retailer is sufficient (c) I consider that the quality of healthy foods offered by my main food retailer is acceptable (d) I consider that the cost of healthy foods offered by my main food retailer is affordable (e) I consider that I have easy access to a food retailer with a good variety of foods near my home (f) I consider the information in the media about food and nutrition positively influences my diet Three items of accessibility to unhealthy foods (g) I consider that fast-food restaurants are easily accessible from my home (h) I consider that fast-food restaurants are easily accessible from my workplace (i) I consider that I have easy access to junk foods at work Response: 5-point agree/disagree Likert scale(the higher score reflects optimal food environments in accessibility on healthy foods and unhealthy foods) Travel time (j) Self-reported travel time from home to the main food retailer by car and on foot Response: less than 10 min, 10–20 min, or more than 20 min. This response was classified binominal variables; less than 10 min (healthy food environments) vs. 10 min or more.	Continuous in Perceived Food Environment Questionnaire and dichotomous in travel time
Caspi et al., 2012 [7]	*Accessibility*	A simplified version of the Neighborhood Environment Walkability Scale [52]. Response: Whether they had a supermarket ‘within walking distance’ of their homes; ‘Yes’ (healthy food environments) vs. ‘No’	Dichotomous
Chapman et al., 2017 [21]	*Affordability* (a, b, c)	Questions relating to perceptions and beliefs about food costs [53,54,55,56,57,58,59]. (a) Perceptions on the affordability: F&V are not affordable in the shop(s) where I buy most of my food’ (b) Perceptions on cost: ‘I sometimes find it difficult to buy F&V for my household because of the cost’ Response (a–b): 5-point agree/disagree Likert scale. Agreed or strongly agreed vs. disagreed or neutral (healthy food environments) (c) Actual cost: how often the cost of F&V meant that their household bought less than they would like. Response (c): 5-point Likert scale. Often or always vs. Never, rarely or sometimes (healthy food environments)	Dichotomous
Flint et al., 2013 [38]	*Availability* (a, c) *Affordability* (e) *Acceptability* (b, d)	Perceived Availability of Health Foods Scale [46] (a) Grocery store choice: There is a good choice of different types of grocery stores in my neighbourhood (b) Grocery store quality: The quality of grocery stores in my neighbourhood is good (c) Choice of F&V: The choice of fresh fruit and vegetables to purchase in my neighbourhood is good (d) Quality of F&V: The quality of fresh fruit and vegetables to purchase in my neighbourhood is good (e) F&V are inexpensive: Fresh fruit and vegetables in my neighbourhood are expensive. Response: 5-point agree/disagree Likert scale (the response of (e) was reverse-coded, and the higher score reflects healthy food environments)	Continuous
Freedman et al., 2019 [35]	*Availability* (a, c) *Acceptability* (b)	Perceptions of healthy food availability [39,43,46,60] (a) A large selection of fruits and vegetables is available in your neighborhood (b) The fresh fruits and vegetables in your neighborhood are of high quality (c) A large selection of low-fat products is available in your neighborhood Response: 4-point agree/disagree Likert scale (the summed higher score reflects healthy food environments) The availability area was defined as a within a 20-min walk or one mile from their home.	Continuous
Gase et al., 2016 [42]	*Acceptability*	The perceived food environment [55,61] In my neighborhood, it is easy for me to find fresh fruits and vegetables. Response: 5-point agree/disagree Likert scale (the higher score reflects healthy food environments)	Continuous
Jilcott Pitts et al., 2015 [36]	*Accessibility* (a) *Availability* (b, c, e, d)	Perceptions of neighborhood barriers [62] Perceived neighborhood nutrition barriers: 5 items (a) Too many fast-food restaurants (b) Not enough food stores with affordable fruits and vegetables (c) Not enough restaurants with healthy food choices (d) Not enough farmer’s markets or fruit stands (e) No place to buy a quick, healthy breakfast to go Response: 5-point Likert scale, not a problem to a big problem, was summed as “perceived neighborhood nutrition barriers” score (ranged 5 to 2; the lower score reflects healthy food environments).	Continuous
Kegler et al., 2014 [29]	*Accessibility* (a) *Availability* (a) *Accommodation* (b)	Neighborhood Environment [47,63] (a) Access to healthy foods in the neighborhood measure: ease of purchase and variety of fruits and vegetables and low-fat products in their neighborhood. (b) Neighborhood social cohesion: whether neighbors were willing to help each other, the neighborhood was close-knit and whether neighbors can be trusted. Response: 5-point agree/disagree Likert scale (the higher score reflects healthy food environments)	Continuous
Liese et al., 2014 [39]	*Accessibility* (d) *Availability* (a, c) *Acceptability* (b)	Perceptions of the Food Environment [43,44,45,46,47,48] The definition of neighborhood was an area within a 20-min walk or about a mile from their home (a) A large selection of fruits and vegetables is available in my neighborhood. (b) The fresh fruits and vegetables in my neighborhood are of high quality. (c) A large selection of low-fat products is available in my neighborhood. Response (a–c): 5-point agree/disagree Likert scale (the higher summed score reflects healthy food environments) (d) How much of a problem would you say that lack of access to adequate food shopping is in your neighborhood? Response (d): 4-point Likert scale; not really a problem, minor problem, somewhat serious problem, or very serious problem (the higher score reflects healthy food environments)	Continuous
Lo et al., 2019 [34]	*Accessibility* (a) *Availability* (b, d, e) *Acceptability* (c, f)	Perceived food environment [47] (a) It is easy to purchase fresh fruits and vegetables in my neighborhood (b) There is a large selection of fresh fruits and vegetables available in my neighborhood (c) The fresh produce in my neighborhood is of high quality (d) It is easy to purchase low-fat products (such as low-fat milk or lean meats) in my neighborhood (e) There is a large selection of low-fat products available in my neighborhood (f) The low-fat products in my neighborhood are of high quality Response: 5-point agree/disagree Likert scale (the summed higher score reflects healthy food environments)	Continuous
Lucan and Mitra, 2012 [25]	*Accessibility* (a, b) *Acceptability* (a, c)	Perceptions of the food environment from 2004 Household Health Survey (Philadelphia Health Management Corporation 2004) [43,44,45] (a) Poor Accessibility of fruits and vegetables: How easy or difficult is it for you to find fruits and vegetables in your neighborhood? Response: 4-point Likert scale. The responses were classified binary variables; difficult/very difficult vs. Easy/very easy (healthy food environments). (b) Poor Accessibility: Do you have to travel outside of your neighborhood to go to a supermarket? Response: Yes vs. No (healthy food environments) (c) Poor Quality: How would you rate the overall quality of groceries available in the stores in your neighborhood? Response: 5-point Likert scale. The responses were classified binary variables; poor (fair/poor/absent) vs. good (excellent/good) (healthy food environments).	Dichotomous
Ma et al., 2018 [40]	*Accessibility* (b) *Availability* (a)	Perceptions of the food environment [43,44,45,46,47] (a) The availability of healthy foods in the neighborhood (range 0–12) (b) Ease of shopping access (range 0–3) (the higher score reflects healthy food environments)	Continuous
Minaker et al., 2013 [37]	*Accessibility* (a, b, c, d, g) *Availability* (e, f) *Acceptability* (k, l) *Affordability* (h, i, j)	Food environment perceptions [7,43,46] (a) There are no food outlets in my neighborhood * (b) It is easy to purchase fresh fruits and vegetables in my neighborhood (c) It is easy to purchase low-fat products (such as low-fat milk or lean meats) in my neighborhood (d) There are a lot of fast-food restaurants in my neighborhood * (e) There is a large selection of fresh fruits and vegetables available in my neighborhood (f) There is a large selection of low-fat products available in my neighborhood (g) It is easy to eat healthily at the restaurants in my neighborhood. (h) I shop elsewhere because the prices in my neighborhood are too high * (i) The produce in my neighborhood is more expensive than that in other neighborhoods * (j) The low-fat products in my neighborhood are more expensive than those in other areas * (k) The fresh produce in my neighborhood is of high quality (l) The low-fat products in my neighborhood are of high quality Response: 4-point agree/disagree Likert scale (the higher summed score reflects healthy food environments). * An item was reverse-scored. The access-related score was integrated accessibility, availability, and acceptability. The score of affordability was used itself as the Food affordability.	Continuous
Oexle et al., 2015 [41]	*Availability*	Perceived availability of neighborhood fast food the Multi-Ethnic Study of Atherosclerosis [46] There are many opportunities to purchase fast foods in my neighborhood (an area within a 20-min walk, or about 1 mile, from their home) such as McDonald’s, Taco Bell, KFC and take-out pizza places, etc. Response: 5-point agree/disagree Likert scale (the higher summed score reflects unhealthy food environments)	Continuous
Sharkey et al., 2010 [32]	*Availability* (a, b, d) *Acceptability* (e) *Affordability* (c, f)	The perceived adequacy of community food resources (a) Little variety in types of foods that can be purchased (b) Few grocery stores or supermarkets (c) Food prices are high. Response: 5-point agree/disagree Likert scale. The responses were classified binary variables; strongly agree/agree vs. others (healthy food environments) Perceptions related to the store where most of the groceries were purchased (d) How would you rate the variety of fruits and vegetables at this store (e) How would you rate the freshness of fruits and vegetables (f) How would you rate the price of fruits and vegetables? Response: 5-point Likert scale. The responses were classified binary variables; fair/poor vs. all others (healthy food environments)	Continuous in community food resources and dichotomous in food store
Springvloet et al., 2014 [30]	*Availability* (a) *Affordability* (b)	Perception of availability in supermarket [64] (a) In the store where I usually do my shopping, there is a sufficient amount of vegetables available Response: 5-point agree/disagree Likert scale (the higher score reflects healthy food environments) Perception of vegetable as being expensive (b) I think eating 200 g of vegetables per day is (select one response below) Response: 5-point Likert scale; very expensive to very cheap (the higher score reflects healthy food environments)	Continuous
Yamaguchi et al., 2019 [33]	*Accessibility*	The perceived availability of food [65,66] How many stores or facilities selling fresh fruits and vegetables are located within one kilometer of your home? Response: 4-point Likert scale, Many to None. The responses were classified binary variables; poor access (few or none) vs. good access (many or some) (healthy food environments)	Dichotomous

F&V: fruits and vegetables. ^a^ Applicable types of perceived food environments were selected from the five types provided below. If there were two or more types, the applicable types were described (i.e., a−l) in the measurement column. *Accessibility*: The location of the food supply source and the ease of getting to that location, counting for travel time and distance. *Availability*: The adequacy of the supply of healthy food; examples in the food environment might include the presence of certain types of restaurants near people’s homes, or the number of places to buy produce. *Affordability*: Food prices and people’s perceptions of worth relative to cost, which is often measured by store audits of specific foods, or regional price indices. *Acceptability*: People’s attitudes about attributes of their local food environment and whether the given supply of products meets their personal standards. *Accommodation*: How well local food sources accept and adapt to the needs of local residents (e.g., store hours and types of payment accepted).

**Table 3:** Measurement tools of dietary habits.

Author, Year	Dietary Habits	Measurements	Variable Type
Alber et al., 2018 [28]	F&V intake	F&V intake The Behavioral Risk Factor Surveillance System [67] Daily Fruit and vegetable consumption (servings/day)	Continuous
Bivoltsis et al., 2020 [26]	F&V intake Diet quality ^a^	F&V intake Fruit and vegetable intakes (servings/day) were rated on a scale ranged from 0 (do not eat) to 5 (6 serves or more). Diet quality The simple RESIDE dietary guideline index or S-RDGI1 [68] The higher score (ranged 0 to 100) reflects a better diet quality using six dietary questionnaires. Healthy diet Healthy component score (range 0 to 12) (the higher score reflects a healthy diet) Unhealthy diet Unhealthy component score (range 0 to 18) (the higher score reflects an unhealthy diet)	Continuous
Carbonneau et al., 2019 [31]	Diet quality	Canadian Healthy Eating Index 2007 [69] C-HEI score (range 0 to 100) was based on the average intake of eight adequacy components and three moderation components from three web-based 24 h recalls using an application (R24W) (Jacques et al., 2016) (the higher score reflects a healthy diet)	Continuous
Caspi et al., 2012 [7]	F&V intake	Prime Screen [70], a brief version of the Semiquantitative Food Frequency Questionnaire The frequency of consumption of six items within the last week (servings/day)	Continuous
Chapman et al., 2017 [21]	F&V intake	F&V intake Estimation of F&V servings based on the Australian Dietary Guidelines [71] How many servings of F&V they consumed each day on average? Do you think the F&V consumption is adequate? Response: too little, about right, too much or not sure. Binary variables were used for the analysis: too little vs. others The above perception of the proper F&V intake was replaced with at least two servings/day of fruit and five servings/day of vegetable.	Dichotomous
Flint et al., 2013 [38]	F&V intake	F&V intake The Block Food Frequency Questionnaire [72,73] 15 items of F&V intake (portions/day) over the past month were calculated	Continuous
Freedman et al., 2019 [35]	Diet quality	Diet quality Healthy Eating Index-2010 scores [74,75] HEI-2010 scores (range 0 to 100) were calculated based on the average of three 24-hour dietary recalls (the higher score reflects a healthy diet)	Dichotomous
Gase et al., 2016 [42]	F&V intake	F&V intake The National Institutes of Health’s Quick Food Scan [76] F&V intake (frequencies/day) was calculated by six items of their frequency of F&V intake in the past seven days. The binominal variable was used for the analysis (no information of the cutoff point).	Dichotomous
Jilcott Pitts et al., 2015 [36]	Diet quality	Diet quality The Dietary Risk Assessment (DRA) (a semi-food frequency questionnaire) [77] A summary score of 4 sub-scales (mean score 27.8) from the DRA (the higher score reflects a healthy diet) (1) nuts, oils, dressings, and spreads, (2) vegetables, fruits, whole grains, and beans, (3) drinks, desserts, snacks, eating out, and salt, and 4) fish, meat, poultry, dairy, and eggs	Continuous
Kegler et al., 2014 [29]	F&V intake Fat intake	F&V intake F&V intake (servings/day) was calculated based on a two-item screener of the food frequency questionnaire [78,79] and the 2005 Behavioral Risk Factor Surveillance System [67] Fat intake Fat intake (% calories) was calculated by the NCI fat screener [80]	Continuous
Liese et al., 2014 [39]	F&V intake	F&V intake F&V intake (servings/day) in the past month was calculated by a food frequency questionnaire from the Multifactor Screener applied in the 2000 National Health Interview Survey [80,81] using a finite number of fruit and vegetable groups (i.e., fruit juice, fruit, lettuce, vegetables, white potatoes, and beans).	Continuous
Lo et al., 2019 [34]	F&V intake	F&V intake F&V intake (servings/day) was calculated based on a food frequency questionnaire from the National Cancer Institute Fruit and Vegetable Screener [82,83] and the average number of cups per day using the 2005 MyPyramid cup equivalents [84].	Continuous
Lucan and Mitra, 2012 [25]	F&V intake Fast-food intake	Two dietary intakes were measured based on the Public Health Management Corporation’s, 2004 Household Health Survey [85] F&V intake How many servings of fruits and vegetables do you eat on a typical day (servings/day)? A serving of a fruit or vegetable is equal to a medium apple, half a cup of peas, or half a large banana Fast-food intake In the past seven days, how many times did you eat food from a fast-food restaurant, such as McDonalds, Pizza Hut or Crown FriedChicken (times/day)?	Continuous
Ma et al., 2018 [40]	F&V intake Diet quality	F&V intake Servings per day was measured [80,81].	Continuous
Minaker et al., 2013 [37]	Diet quality	Diet quality Healthy Eating Index adapted for Canada (HEI-C) scores [69] Mean HEI-C scores over two days were calculated by diet record data (range 0 to 100; the higher score reflects a healthy diet).	Continuous
Oexle et al., 2015 [41]	Fast-food intake	Fast-food intake A slightly altered question from the Multi-Ethnic Study of Atherosclerosis [45,46] How often do you [typically] eat a meal from a fast-food place such as McDonalds’s, KFC, Taco Bell or take-out pizza places? By meal we mean breakfast, lunch or dinner, include eat in or takeout. The frequency was classified binary variables, 1 time/week vs. never and <1 time/week vs. never	Dichotomous
Sharkey et al., 2010 [32]	F&V intake	F&V intake Fruit and vegetable intakes were separately measured by self-reported two-item screener [86,87]. (1) The number of servings of fruit (1/2 cup of fruit or 3/4 cups fruit juice) usually consumed each day (2) The number of servings of vegetables (1/2 cup cooked or 1 cup raw) consumed daily. Total fruit and vegetable intakes were calculated by combining (1) and (2).	Continuous
Springvloet et al., 2014 [30]	Vegetable intake	Vegetable intake Food frequency questionnaire [88,89] Four items using a reference period of one month (g/day) (1) How many days per week they usually consume cooked and raw vegetables or salads (ranging from 0 to 7 days per week)? (2) How many tablespoons of cooked and raw vegetables or salads they usually ate on these days (ranging from one to six or more)?	Continuous
Yamaguchi et al., 2019 [33]	F&V intake Meat and fish intake	F&V intake and Meat and fish intake Average intake of vegetables/fruits and meat/fish over a one-month (times/day) [65] was calculated by the response of ‘every day and over twice/day, every day and once/day, 4–6 times/week, 2–3 times/week, once-a-week, less than once-a-week, or almost never’.	Continuous

F&V: fruits and vegetables. ^a^ Diet quality was assessed by scores based on an indicator.

**Table 4:** Findings and statistical analyses of the association of perceived food environments with dietary food or habits.

Author, Year	Findings *β* Coefficient (SE) or 95%CI) or OR (95%CI)	Association ^a^		Covariates	Statistical Analyses
		Healthy Food or Diets	Unhealthy Food or Diets		
Alber et al., 2018 [28]	*Accessibility* Ease of purchasing in neighborhood *β* (SE) = −0.02 (0.16)	N.S. (negative)		Age, sex, race/ethnicity, income, education and home food environment	Multiple linear regression model
	*Availability* Availability in neighborhood *β* (SE) = −0.21 (0.13) *	Negative			
	*Affordability* Price in neighborhood *β* (SE) = −0.08 (0.16)	N.S. (negative)			
	*Acceptability* Quality in neighborhood *β* (SE) = 0.33 (0.14) **	Positive			
Bivoltsis et al., 2020 [26]	*Accessibility* Presence of a café or restaurant within 15 min walk of home Decrease (i.e., yes to no: improved healthy perceived food environments) *β* (95%CI) = 0.003 (−0.15, 0.16) for healthy dietary score	N.S. (positive)		All baseline participant characteristics, baseline diet, time between baseline and follow-up questionnaire completion, self-selection variables and accounting for clustering in the 73 new developments	Mixed linear model (the change from baseline [before moving house] to follow-up [1–2 years after relocation])
	*β* (95%CI) = 0.02 (−0.12, 0.16) for F&V intake	N.S. (positive)			
	*β* (95%CI) = 0.01 (−0.23, 0.25) for unhealthy dietary score		N.S. (negative)		
	Increase (i.e., no to yes: worsened unhealthy perceived food environments) *β* (95%CI) = 0.07 (−0.15, 0.28) for healthy dietary score	N.S. (negative)			
	*β* (95%CI) = 0.02 (−0.17, 0.21) for F&V intake	N.S. (negative)			
	*β* (95%CI) = 0.41 (0.08, 0.73) * for unhealthy dietary score		Positive		
	*Accessibility* Presence of a supermarket/greengrocer within 15 min walk of home Decrease (i.e., yes to no: worsened unhealthy perceived food environments) *β* (95%CI) = 0.06 (−0.08, 0.21) for healthy dietary score	N.S. (negative)			
	*β* (95%CI) = 0.05 (−0.08, 0.18) for F&V intake	N.S. (negative)			
	*β* (95%CI) = 0.15 (−0.07, 0.38) for unhealthy dietary score		N.S. (positive)		
	Increase (i.e., no to yes: improved healthy perceived food environments) *β* (95%CI) = 0.05 (−0.20, 0.30) for healthy dietary score	N.S. (positive)			
	*β* (95%CI) = 0.05 (−0.18, 0.27) for F&V intake	N.S. (positive)			
	*β* (95%CI) = 0.40 (0.02, 0.79) * for unhealthy dietary score		Negative		
Carbonneau et al., 2019 [31]	*Accessibility* Travel time from home to the main retailer *β* (95%CI) = 1.31 (−0.62, 3.24)	N.S. (positive)		Sex, age groups, education, household annual income, marital status, smoking status, nutrition knowledge and reporting status of dietary intake	Multiple linear regression model
	*Accessibility*, *availability, affordability, acceptability*, and *accommodation* Perceived accessibility to healthy foods *β* (95%CI) = 0.01 (−1.51, 1.53)	N.S. (positive)			
Caspi et al., 2012 [7]	*Accessibility* Perceived supermarket access *β* (SE) = 0.48 (0.12) ***	Positive		Weekly income, country of origin, age, gender, food insecurity and town of residence	Generalized estimating equation
Chapman et al., 2017 [21]	*Affordability* ‘F&V are not affordable in the shop(s) where I buy most of my food’ Agree (vs. disagree/neutral) OR (95% CI) = 0.77 (0.63, 0.95) * for meeting fruits recommendation (too little [< 2 servings/day] vs. others)	Positive		Age, sex, remoteness of place of residence, socio-economic quintile of advantage/disadvantage, education, household income and number of children	Multivariable logistic regression model
	Agree (vs. disagree/neutral) OR (95% CI) = 0.85 (0.59, 1.22) for meeting vegetable recommendation (too little [< 5 servings/day] vs. others)	N.S. (positive)			
	I sometimes find it difficult to buy F&V for my household because of the cost Agree (vs. disagree/neutral) OR (95% CI) = 0.84 (0.70, 0.99) * for meeting fruits recommendation (too little vs. others)	Positive			
	Agree (vs. disagree/neutral) OR (95% CI) = 0.82 (0.61, 1.10) for meeting vegetable recommendation (too little vs. others)	N.S. (positive)			
	The cost of F&V means that my household buys less than I would like Often (vs. sometimes) OR (95% CI) = 0.61 (0.50, 0.75) ** for meeting fruits recommendation (too little vs. others)	Positive			
	Often (vs. sometimes) OR (95% CI) = 0.84 (0.59, 1.19) for meeting vegetable recommendation (too little vs. others)	N.S. (positive)			
Flint et al., 2013 [38]	*Availability* Choice of F&V *β* = 0.03	N.S. (positive)		Age, sex, race/ethnicity, presence of children under 12 in the household, household income, completed secondary education, employment status and mode of transport for food shopping	Linear regression model
	Grocery store choice *β* = −0.03	N.S. (negative)			
	*Affordability* F&V are inexpensive *β* = 0.04	N.S. (positive)			
	*Acceptability* Grocery store quality *β* = −0.03	N.S. (negative)			
	Quality of F&V *β* = 0.01	N.S. (positive)			
Freedman et al., 2019 [35]	*Availability* and *acceptability* Perception of healthy food availability low-income communities in Cleveland *β* = no direct association in the path model	N.S. (–)		Income, race and sex	Two path analyses: Cleveland model and the Columbus model
	low-income communities in Columbus *β* = −0.13 *	Negative			
Gase et al., 2016 [42]	*Acceptability* Perceived ease of accessing fruit and vegetable scale Incident Rate Ratio (95% CI) = 1.05 (1.01, 1.09) *	Positive		Age, gender, race/ethnicity and education level	Negative binomial regression model
Jilcott Pitts et al., 2015 [36]	*Accessibility* and *availability* Perceived neighborhood nutrition barriers *β* (SE) = −0.13 (0.05) *	Positive		Age at enrollment, race, sex and education level	Multiple linear regression model
Kegler et al., 2014 [29]	*Accessibility* and *availability* Neighborhood access to healthy foods *β* (SE) = 0.04 (0.04) for F&V intake	N.S. (positive)		–	Path analysis, a form of structural equation model
	*β* (SE) = 0.04 (0.04) for fat intake		N.S. (positive)		
	*Accommodation* Neighborhood social cohesion *β* (SE) = −0.01 (0.06) for F&V intake	N.S. (negative)			
	*β* (SE) = 0.09 (0.06) for fat intake		N.S. (positive)		
Liese et al., 2014 [39]	*Accessibility* Ease of Shopping Access *β* = 0.01	N.S. (positive)		–	Path analysis
	*Availability* and *acceptability* Supermarket Availability *β* = 0.08 *	Positive			
Lo et al., 2019 [34]	*Accessibility* and *availability* *β* (SE) = 0.14 (0.13)	N.S. (positive)		Age, body mass index, marital status and education	Linear regression model
Lucan and Mitra, 2012 [25]	*Accessibility* Poor *accessibility* of fruits and vegetables IRR (95%CI) = 0.99 (0.93, 1.06) for F&V intake	N.S. (positive)		The corresponding contextual variable at the neighborhood level, individual-level sociodemographic, and neighborhood sociodemographic	Poisson regression and logistic regression models
	IRR (95%CI) = 1.31 (1.19, 1.45) ** for fast-food intake		Positive		
	Poor supermarket *accessibility* IRR (95%CI) = 1.01 (0.98, 1.04) for F&V intake	N.S. (negative)			
	IRR (95%CI) = 1.06 (1.00, 1.11) * for fast-food intake		Positive		
	*Acceptability* Poor grocery quality IRR (95%CI) = 1.01 (0.97, 1.05) for F&V intake	N.S. (negative)			
	IRR (95%CI) = 1.20 (1.12, 1.28) ** for fast-food intake		Positive		
Ma et al., 2018 [40]	*Accessibility* Ease of shopping access *β* = no direct association in the path model	N.S.		–	Path analysis
	*Availability* Availability of healthy Foods *β* = no direct association in the path model	N.S.			
Minaker et al., 2013 [37]	*Accessibility*, *availability,* and *acceptability* Access-related *β* (SE) = 0.17 (0.47) in women	N.S. (positive)		Age, education level, household income level, car ownership and waist circumference	Multilevel linear regression model
	*β* (SE) = 1.09 (0.46) * in men	Positive			
	*Affordability* Food affordability *β* (SE) = 0.24 (0.49) in women	N.S. (positive)			
	*β* (SE) = 0.31 (0.46) in men	N.S. (positive)			
Oexle et al., 2015 [41]	*Availability* Perceived availability of fast food OR (95%CI) = 1.20 (0.80, 1.79) for fast-food consumption 1 time/week (vs. never)		N.S. (positive)	Age, sex, race/ethnicity, level of education, employment status and urbanity of living environment	Multinomial logistic regression model
	OR (95%CI) = 1.30 (0.88, 1.92) for fast-food consumption < 1 time/week (vs. never)		N.S. (positive)		
Sharkey et al., 2010 [32]	*Availability*, *acceptability,* and *affordability* The perceived adequacy of community food resources Food not last *β* (SE) = −0.97 (0.18) ***	Positive		Individual characteristics (live alone, female and age) and distance to nearest food store (Supermarket)	Multivariable linear regression model
	Few grocery stores *β* (SE) = −0.30 (0.13) *	Positive			
	Fruit/vegetable (little) variety *β* (SE) = −0.40 (0.20) *	Positive			
Springvloet et al., 2014 [30]	*Availability* Perception of availability in supermarket *β* = −0.05 *	Negative		Age, sex, place of residence, ethnicity and education	Linear regression model
	*Affordability* Perception whether vegetables are expensive *β* = −0.05 *	Negative			
Yamaguchi et al., 2019 [33]	*Accessibility* Poor access (vs. good access) *β* (SE) = −0.09 (0.01) *** for V&F intake	Positive		Age, sex, family structure, BMI, marital status, activities of daily living, the number of remaining teeth, presence of comorbidities, smoking status, household income, and years of schooling	Multilevel logistic regression model
	*β* (SE) = −0.03 (0.004) *** for Meat & fish intake	Positive			

SE: standard error, OR: odds ratio, CI: confidential interval, IRR: incident rate ratio, F&V: fruits and vegetables, Positive or Negative: direction of the significant association, N.S. (negative or positive): no significance (the direction of the association): no information. Statistically significant associations: * *p* < 0.05, ** *p* < 0.01, and *** *p* < 0.001. ^a^ A “positive” association existed when that healthy perceived food environments were significantly associated with a higher intake of healthy food or a lower intake of unhealthy food, and “negative” association, when healthy perceived food environments were significantly associated with a lower intake of healthy food or a higher intake of unhealthy food. The “positive” association indicated instances when unhealthy perceived food environments were significantly associated with a lower intake of healthy food or a higher intake of unhealthy food, and “negative” indicated instances when unhealthy perceived food environments were significantly associated with a higher intake of healthy food or a lower intake of unhealthy food.

**Table 5:** The frequencies at which the 19 studies extracted food access dimensions in their analyses and significant association between dimensions and healthy food or diets.

Food Access Dimensions	Studies	*N* ^a^	Positive ^b^	Negative ^c^
*Accessibility*	Alber et al., 2018 [28]; Bivoltsis et al., 2020 [26]; Carbonneau et al., 2019 [31]; Caspi et al., 2012 [7] *^b^; Lucan and Mitra, 2012 [25]; Lo et al., 2019 [34]; Ma et al., 2018 [40]; Yamaguchi et al., 2019 [33] *^b^	8	2	–
*Availability*	Alber et al., 2018 [28] *^c^; Flint et al., 2013 [38]; Ma et al., 2018 [40]; Oexle et al., 2015 [41]; Springvloet et al., 2014 [30] *^c^	5	–	2
*Affordability*	Alber et al., 2018 [28]; Chapman et al., 2017 [21] *^b^; Flint et al., 2013 [38]; Minaker et al., 2013 [37]; Springvloet et al., 2014 [30] *^c^	5	1	1
*Acceptability*	Alber et al., 2018 [28] *^b^; Flint et al., 2013 [38]; Gase et al., 2016 [42] *^b^; Lucan and Mitra, 2012 [25]	4	2	–
*Accommodation*	Kegler et al., 2014 [29]	1	–	–
*Accessibility* and *availability*	Jilcott Pitts et al., 2015 [36] *^b^; Kegler et al., 2014 [29]	2	1	–
*Availability* and *acceptability*	Freedman et al., 2019 [35] *^c^; Liese et al., 2014 [39] *^b^	2	1	1
*Accessibility, availability,* and *acceptability*	Minaker et al., 2013 [37] *^b^; Lo et al., 2019 [34]	2	1	–
*Availability, acceptability,* and *affordability*	Sharkey et al., 2010 [32] *^b^	1	1	–
*Accessibility, availability, affordability, acceptability,* and *accommodation*	Carbonneau et al., 2019 [31]	1	–	–

*^b^ Significant positive association. *^c^ Significant negative association. ^a^ Number of studies in each dimension. ^b^ Number of studies showing significant positive associations of each dimension with healthy food or diets. ^c^ Number of studies showing significant negative associations of each dimension with healthy food or diets.

## Data Availability

Not applicable.

## Supplementary Materials

The following supporting information can be downloaded at: https://www.mdpi.com/article/10.3390/nu14091788/s1, Table S1: Keyword search conducted on Pub Med; Table S2: Keyword search conducted on the Web of Science; Table S3: Assessment sheet of the risk of bias; and Table S4: Results of the risk of bias assessment.

## References

[1] Department of Economic and Social Affairs, Sustainable Development, United Nations Goals 2. End Hunger, Achieve Food Security and Improved Nutrition and Promote Sustainable Agriculture. https://sdgs.un.org/goals/goal2.

[2] Downs S.M., Ahmed S., Fanzo J., Herforth A. (2020). Food Environment Typology: Advancing an Expanded Definition, Framework, and Methodological Approach for Improved Characterization of Wild, Cultivated, and Built Food Environments toward Sustainable Diets. Foods.

[3] Food and Agriculture Organization of the United Nations and World Health Organization Sustainable Healthy Diets–Guiding Principles. http://www.fao.org/3/ca6640en/ca6640en.pdf.

[4] Cummins S., Macintyre S. (2002). “Food deserts”—Evidence and Assumption in Health Policy Making. BMJ (Clin. Res. Ed.).

[5] Black C., Moon G., Baird J. (2014). Dietary inequalities: What is the evidence for the effect of the neighbourhood food environment?. Health Place.

[6] Lytle L.A., Sokol R.L. (2017). Measures of the food environment: A systematic review of the field, 2007–2015. Health Place.

[7] Caspi C.E., Kawachi I., Subramanian S.V., Adamkiewicz G., Sorensen G. (2012). The relationship between diet and perceived and objective access to supermarkets among low-income housing residents. Soc. Sci. Med..

[8] Aggarwal A., Cook A.J., Jiao J., Seguin R.A., Vernez Moudon A., Hurvitz P.M., Drewnowski A. (2014). Access to supermarkets and fruit and vegetable consumption. Am. J. Public Health.

[9] Morland K., Filomena S. (2008). The utilization of local food environments by urban seniors. Prev. Med..

[10] Penchansky R., Thomas J.W. (1981). The concept of access: Definition and relationship to consumer satisfaction. Med. Care.

[11] Glanz K., Sallis J.F., Saelens B.E., Frank L.D. (2005). Healthy nutrition environments: Concepts and measures. Am. J. Health Promot..

[12] Caspi C.E., Sorensen G., Subramanian S.V., Kawachi I. (2012). The local food environment and diet: A systematic review. Health Place.

[13] Moher D., Liberati A., Tetzlaff J., Altman D.G. (2009). Preferred reporting items for systematic reviews and meta-analyses: The PRISMA statement. BMJ (Clin. Res. Ed.).

[14] Cobb L.K., Appel L.J., Franco M., Jones-Smith J.C., Nur A., Anderson C.A. (2015). The relationship of the local food environment with obesity: A systematic review of methods, study quality, and results. Obesity (Silver Spring Md.).

[15] The World Bank Group Country Income Classifications for the World Bank’s 2020 Fiscal Year. https://datahelpdesk.worldbank.org/knowledgebase/articles/906519-world-bank-country-and-lending-groups.

[16] Ouzzani M., Hammady H., Fedorowicz Z., Elmagarmid A. (2016). Rayyan-a web and mobile app for systematic reviews. Syst. Rev..

[17] Bero L., Chartres N., Diong J., Fabbri A., Ghersi D., Lam J., Lau A., McDonald S., Mintzes B., Sutton P. (2018). The risk of bias in observational studies of exposures (ROBINS-E) tool: Concerns arising from application to observational studies of exposures. Syst. Rev..

[18] Hörnell A., Berg C., Forsum E., Larsson C., Sonestedt E., Åkesson A., Lachat C., Hawwash D., Kolsteren P., Byrnes G. (2017). Perspective: An Extension of the STROBE Statement for Observational Studies in Nutritional Epidemiology (STROBE-nut): Explanation and Elaboration. Adv. Nutr. (Bethesda Md.).

[19] Wells G.A., Shea B., O’Connell D., Peterson J., Welch V., Losos M., Tugwell P. The Newcastle-Ottawa Scale (NOS) for Assessing the Quality of Nonrandomised Studies in Meta-Analyses. http://www.ohri.ca/programs/clinical_epidemiology/oxford.asp.

[20] Fletcher H.R., Fletcher S.W., Fletcher S.G. (2014). Clinical Epidemiology: The Essentials.

[21] Chapman K., Goldsbury D., Watson W., Havill M., Wellard L., Hughes C., Bauman A., Allman-Farinelli M. (2017). Exploring perceptions and beliefs about the cost of fruit and vegetables and whether they are barriers to higher consumption. Appetite.

[22] Menezes M.C., Diez Roux A.V., Souza Lopes A.C. (2018). Fruit and vegetable intake: Influence of perceived food environment and self-efficacy. Appetite.

[23] Duran A.C., de Almeida S.L., Latorre Mdo R., Jaime P.C. (2016). The role of the local retail food environment in fruit, vegetable and sugar-sweetened beverage consumption in Brazil. Public Health Nutr..

[24] Lucan S.C., Hillier A., Schechter C.B., Glanz K. (2014). Objective and self-reported factors associated with food-environment perceptions and fruit-and-vegetable consumption: A multilevel analysis. Prev. Chronic Dis..

[25] Lucan S.C., Mitra N. (2012). Perceptions of the food environment are associated with fast-food (not fruit-and-vegetable) consumption: Findings from multi-level models. Int. J. Public Health.

[26] Bivoltsis A., Trapp G., Knuiman M., Hooper P., Ambrosini G.L. (2020). The influence of the local food environment on diet following residential relocation: Longitudinal results from RESIDential Environments (RESIDE). Public Health Nutr..

[27] Trapp G.S., Hickling S., Christian H.E., Bull F., Timperio A.F., Boruff B., Shrestha D., Giles-Corti B. (2015). Individual, Social, and Environmental Correlates of Healthy and Unhealthy Eating. Health Educ. Behav..

[28] Alber J.M., Green S.H., Glanz K. (2018). Perceived and Observed Food Environments, Eating Behaviors, and BMI. Am. J. Prev. Med..

[29] Kegler M.C., Swan D.W., Alcantara I., Feldman L., Glanz K. (2014). The influence of rural home and neighborhood environments on healthy eating, physical activity, and weight. Prev. Sci..

[30] Springvloet L., Lechner L., Oenema A. (2014). Can individual cognitions, self-regulation and environmental variables explain educational differences in vegetable consumption?: A cross-sectional study among Dutch adults. Int. J. Behav. Nutr. Phys. Act.

[31] Carbonneau E., Lamarche B., Robitaille J., Provencher V., Desroches S., Vohl M.C., Begin C., Belanger M., Couillard C., Pelletier L. (2019). Social Support, but Not Perceived Food Environment, Is Associated with Diet Quality in French-Speaking Canadians from the PREDISE Study. Nutrients.

[32] Sharkey J.R., Johnson C.M., Dean W.R. (2010). Food Access and Perceptions of the Community and Household Food Environment as Correlates of Fruit and Vegetable Intake among Rural Seniors. BMC Geriatr..

[33] Yamaguchi M., Takahashi K., Hanazato M., Suzuki N., Kondo K., Kondo N. (2019). Comparison of Objective and Perceived Access to Food Stores Associated with Intake Frequencies of Vegetables/Fruits and Meat/Fish among Community-Dwelling Older Japanese. Int. J. Environ. Res. Public Health.

[34] Lo B.K., Loui C., Folta S.C., Flickinger A., Connor L.M., Liu E., Megiel S., Seguin R.A. (2019). Self-efficacy and cooking confidence are associated with fruit and vegetable intake in a cross-sectional study with rural women. Eat. Behav..

[35] Freedman D.A., Bell B.A., Clark J.K., Sharpe P.A., Trapl E.S., Borawski E.A., Pike S.N., Rouse C., Sehgal A.R. (2019). Socioecological Path Analytic Model of Diet Quality among Residents in Two Urban Food Deserts. J. Acad. Nutr. Diet..

[36] Jilcott Pitts S.B., Keyserling T.C., Johnston L.F., Smith T.W., McGuirt J.T., Evenson K.R., Rafferty A.P., Gizlice Z., Garcia B.A., Ammerman A.S. (2015). Associations between neighborhood-level factors related to a healthful lifestyle and dietary intake, physical activity, and support for obesity prevention polices among rural adults. J. Community Health.

[37] Minaker L.M., Raine K.D., Wild T.C., Nykiforuk C.I., Thompson M.E., Frank L.D. (2013). Objective food environments and health outcomes. Am. J. Prev. Med..

[38] Flint E., Cummins S., Matthews S. (2013). Do perceptions of the neighbourhood food environment predict fruit and vegetable intake in low-income neighbourhoods?. Health Place.

[39] Liese A.D., Bell B.A., Barnes T.L., Colabianchi N., Hibbert J.D., Blake C.E., Freedman D.A. (2014). Environmental influences on fruit and vegetable intake: Results from a path analytic model. Public Health Nutr..

[40] Ma X.N., Blake C.E., Barnes T.L., Bell B.A., Liese A.D. (2018). What does a person’s eating identity add to environmental influences on fruit and vegetable intake?. Appetite.

[41] Oexle N., Barnes T.L., Blake C.E., Bell B.A., Liese A.D. (2015). Neighborhood fast food availability and fast food consumption. Appetite.

[42] Gase L.N., Glenn B., Kuo T. (2016). Self-Efficacy as a Mediator of the Relationship Between the Perceived Food Environment and Healthy Eating in a Low Income Population in Los Angeles County. J. Immigr. Minor. Health.

[43] Moore L.V., Diez Roux A.V., Brines S. (2008). Comparing Perception-Based and Geographic Information System (GIS)-based characterizations of the local food environment. J. Urban Health.

[44] Moore L.V., Diez Roux A.V., Nettleton J.A., Jacobs D.R. (2008). Associations of the local food environment with diet quality—A comparison of assessments based on surveys and geographic information systems: The multi-ethnic study of atherosclerosis. Am. J. Epidemiol..

[45] Moore L.V., Diez Roux A.V., Nettleton J.A., Jacobs D.R., Franco M. (2009). Fast-food consumption, diet quality, and neighborhood exposure to fast food: The multi-ethnic study of atherosclerosis. Am. J. Epidemiol..

[46] Mujahid M.S., Diez Roux A.V., Morenoff J.D., Raghunathan T. (2007). Assessing the measurement properties of neighborhood scales: From psychometrics to ecometrics. Am. J. Epidemiol..

[47] Echeverria S.E., Diez-Roux A.V., Link B.G. (2004). Reliability of self-reported neighborhood characteristics. J. Urban Health.

[48] Ma X., Barnes T.L., Freedman D.A., Bell B.A., Colabianchi N., Liese A.D. (2013). Test-retest reliability of a questionnaire measuring perceptions of neighborhood food environment. Health Place.

[49] Green S.H., Glanz K. (2015). Development of the Perceived Nutrition Environment Measures Survey. Am. J. Prev. Med..

[50] Cerin E., Saelens B.E., Sallis J.F., Frank L.D. (2006). Neighborhood Environment Walkability Scale: Validity and development of a short form. Med. Sci. Sports Exerc..

[51] Carbonneau E., Robitaille J., Lamarche B., Corneau L., Lemieux S. (2017). Development and validation of the Perceived Food Environment Questionnaire in a French-Canadian population. Public Health Nutr..

[52] Saelens B.E., Sallis J.F., Black J.B., Chen D. (2003). Neighborhood-based differences in physical activity: An environment scale evaluation. Am. J. Public Health.

[53] Anderson J.V., Bybee D.I., Brown R.M., McLean D.F., Garcia E.M., Breer M.L., Schillo B.A. (2001). 5 a day fruit and vegetable intervention improves consumption in a low income population. J. Am. Diet. Assoc..

[54] Bihan H., Castetbon K., Mejean C., Peneau S., Pelabon L., Jellouli F., Le Clesiau H., Hercberg S. (2010). Sociodemographic factors and attitudes toward food affordability and health are associated with fruit and vegetable consumption in a low-income French population. J. Nutr..

[55] Dibsdall L.A., Lambert N., Bobbin R.F., Frewer L.J. (2003). Low-income consumers’ attitudes and behaviour towards access, availability and motivation to eat fruit and vegetables. Public Health Nutr..

[56] Giskes K., van Lenthe F.J., Kamphuis C.B., Huisman M., Brug J., Mackenbach J.P. (2009). Household and food shopping environments: Do they play a role in socioeconomic inequalities in fruit and vegetable consumption? A multilevel study among Dutch adults. J. Epidemiol. Community Health.

[57] Inglis V., Ball K., Crawford D. (2008). Socioeconomic variations in women’s diets: What is the role of perceptions of the local food environment?. J. Epidemiol. Community Health.

[58] Mushi-Brunt C., Haire-Joshu D., Elliott M. (2007). Food spending behaviors and perceptions are associated with fruit and vegetable intake among parents and their preadolescent children. J. Nutr. Educ. Behav..

[59] Yeh M.C., Ickes S.B., Lowenstein L.M., Shuval K., Ammerman A.S., Farris R., Katz D.L. (2008). Understanding barriers and facilitators of fruit and vegetable consumption among a diverse multi-ethnic population in the USA. Health Promot. Int..

[60] Gustafson A.A., Sharkey J., Samuel-Hodge C.D., Jones-Smith J., Folds M.C., Cai J., Ammerman A.S. (2011). Perceived and objective measures of the food store environment and the association with weight and diet among low-income women in North Carolina. Public Health Nutr..

[61] Caldwell E.M., Miller Kobayashi M., DuBow W.M., Wytinck S.M. (2009). Perceived access to fruits and vegetables associated with increased consumption. Public Health Nutr..

[62] Jilcott S.B., Keyserling T.C., Samuel-Hodge C.D., Rosamond W., Garcia B., Will J.C., Farris R.P., Ammerman A.S. (2006). Linking clinical care to community resources for cardiovascular disease prevention: The North Carolina Enhanced WISEWOMAN project. J. Womens Health.

[63] Sampson R.J., Raudenbush S.W., Earls F. (1997). Neighborhoods and violent crime: A multilevel study of collective efficacy. Science.

[64] Springvloet L., Lechner L., Oenema A. (2014). Planned development and evaluation protocol of two versions of a web-based computer-tailored nutrition education intervention aimed at adults, including cognitive and environmental feedback. BMC Public Health.

[65] Nakamura H., Nakamura M., Okada E., Ojima T., Kondo K. (2017). Association of food access and neighbor relationships with diet and underweight among community-dwelling older Japanese. J. Epidemiol..

[66] Tani Y., Suzuki N., Fujiwara T., Hanazato M., Kondo N., Miyaguni Y., Kondo K. (2018). Neighborhood food environment and mortality among older Japanese adults: Results from the JAGES cohort study. Int. J. Behav. Nutr. Phys. Act..

[67] Centers for Disease Control and Prevention Behavioral Risk Factor Surveillance System. https://www.cdc.gov/brfss/index.html.

[68] Bivoltsis A., Trapp G.S.A., Knuiman M., Hooper P., Ambrosini G.L. (2018). Can a Simple Dietary Index Derived from a Sub-Set of Questionnaire Items Assess Diet Quality in a Sample of Australian Adults?. Nutrients.

[69] Garriguet D. (2009). Diet quality in Canada. Health Rep..

[70] Rifas-Shiman S.L., Willett W.C., Lobb R., Kotch J., Dart C., Gillman M.W. (2001). PrimeScreen, a brief dietary screening tool: Reproducibility and comparability with both a longer food frequency questionnaire and biomarkers. Public Health Nutr..

[71] National Health and Medical Research Council (2013). Australian Dietary Guidelines.

[72] Block G., Hartman A.M., Dresser C.M., Carroll M.D., Gannon J., Gardner L. (1986). A data-based approach to diet questionnaire design and testing. Am. J. Epidemiol..

[73] Michels K.B., Giovannucci E., Chan A.T., Singhania R., Fuchs C.S., Willett W.C. (2006). Fruit and vegetable consumption and colorectal adenomas in the Nurses’ Health Study. Cancer Res..

[74] Guenther P.M., Casavale K.O., Reedy J., Kirkpatrick S.I., Hiza H.A., Kuczynski K.J., Kahle L.L., Krebs-Smith S.M. (2013). Update of the Healthy Eating Index: HEI-2010. J. Acad. Nutr. Diet..

[75] U.S. Department of Agriculture, U.S. Department of Health and Human Services (2010). Dietary Guidelines for Americans.

[76] National Cancer Institute Eating at America’s Table Study: Quick food Scan. http://riskfactor.cancer.gov/diet/screeners/fruitveg/allday.pdf.

[77] Ammerman A.S., Haines P.S., DeVellis R.F., Strogatz D.S., Keyserling T.C., Simpson R.J., Siscovick D.S. (1991). A brief dietary assessment to guide cholesterol reduction in low-income individuals: Design and validation. J. Am. Diet. Assoc..

[78] Gattshall M.L., Shoup J.A., Marshall J.A., Crane L.A., Estabrooks P.A. (2008). Validation of a survey instrument to assess home environments for physical activity and healthy eating in overweight children. Int. J. Behav. Nutr. Phys. Act..

[79] Serdula M., Coates R., Byers T., Mokdad A., Jewell S., Chávez N., Mares-Perlman J., Newcomb P., Ritenbaugh C., Treiber F. (1993). Evaluation of a brief telephone questionnaire to estimate fruit and vegetable consumption in diverse study populations. Epidemiology.

[80] Thompson F.E., Midthune D., Subar A.F., Kahle L.L., Schatzkin A., Kipnis V. (2004). Performance of a short tool to assess dietary intakes of fruits and vegetables, percentage energy from fat and fibre. Public Health Nutr..

[81] Thompson F.E., Midthune D., Subar A.F., McNeel T., Berrigan D., Kipnis V. (2005). Dietary intake estimates in the National Health Interview Survey, 2000: Methodology, results, and interpretation. J. Am. Diet. Assoc..

[82] Subar A.F., Thompson F.E., Kipnis V., Midthune D., Hurwitz P., McNutt S., McIntosh A., Rosenfeld S. (2001). Comparative validation of the Block, Willett, and National Cancer Institute food frequency questionnaires: The Eating at America’s Table Study. Am. J. Epidemiol..

[83] Thompson F.E., Kipnis V., Subar A.F., Krebs-Smith S.M., Kahle L.L., Midthune D., Potischman N., Schatzkin A. (2000). Evaluation of 2 brief instruments and a food-frequency questionnaire to estimate daily number of servings of fruit and vegetables. Am. J. Clin. Nutr..

[84] National Cancer Institute Fruit & Vegetable Screeners in the Eating at America’s Table Study (EATS): Scoring. https://epi.grants.cancer.gov/diet/screeners/fruitveg/scoring/.

[85] Philadelphia Health Management Corporation Community Health Data Base-Southeastern Pennsylvania Household Health Survey. https://www.phmc.org/site/index.php?option=com_content&view=article&id=66&Itemid=20.

[86] Campbell M.K., Carr C., Devellis B., Switzer B., Biddle A., Amamoo M.A., Walsh J., Zhou B., Sandler R. (2009). A randomized trial of tailoring and motivational interviewing to promote fruit and vegetable consumption for cancer prevention and control. Ann. Behav. Med..

[87] Resnicow K., Odom E., Wang T., Dudley W.N., Mitchell D., Vaughan R., Jackson A., Baranowski T. (2000). Validation of three food frequency questionnaires and 24-hour recalls with serum carotenoid levels in a sample of African-American adults. Am. J. Epidemiol..

[88] Bogers R.P., Van Assema P., Kester A.D., Westerterp K.R., Dagnelie P.C. (2004). Reproducibility, validity, and responsiveness to change of a short questionnaire for measuring fruit and vegetable intake. Am. J. Epidemiol..

[89] Van Assema P., Brug J., Ronda G., Steenhuis I., Oenema A. (2002). A short dutch questionnaire to measure fruit and vegetable intake: Relative validity among adults and adolescents. Nutr. Health.

[90] Hermstad A.K., Swan D.W., Kegler M.C., Barnette J.K., Glanz K. (2010). Individual and environmental correlates of dietary fat intake in rural communities: A structural equation model analysis. Soc. Sci. Med..

[91] Zobel E.H., Hansen T.W., Rossing P., von Scholten B.J. (2016). Global Changes in Food Supply and the Obesity Epidemic. Curr. Obes. Rep..

[92] Barnidge E.K., Radvanyi C., Duggan K., Motton F., Wiggs I., Baker E.A., Brownson R.C. (2013). Understanding and addressing barriers to implementation of environmental and policy interventions to support physical activity and healthy eating in rural communities. J. Rural Health.

[93] Earnshaw V.A., Karpyn A. (2020). Understanding stigma and food inequity: A conceptual framework to inform research, intervention, and policy. Transl. Behav. Med..

[94] Dumas-Mallet E., Button K.S., Boraud T., Gonon F., Munafò M.R. (2017). Low statistical power in biomedical science: A review of three human research domains. R. Soc. Open Sci..

[95] Budhiraja P., Kaplan B., Mustafa R.A. (2020). Handling of Missing Data. Transplantation.

[96] Menezes M.C., Diez Roux A.V., Costa B.V.L., Lopes A.C.S. (2018). Individual and food environmental factors: Association with diet. Public Health Nutr..

[97] Okechukwu C., Davison K., Emmons K., Barkman L., Kawachi I., Glymour M.M. (2014). Changing Health Behaviors in a Social Context. Social Epidemiology.

[98] Van der Horst K., Bucher T., Duncanson K., Murawski B., Labbe D. (2019). Consumer Understanding, Perception and Interpretation of Serving Size Information on Food Labels: A Scoping Review. Nutrients.

[99] Middleton G., Keegan R., Smith M.F., Alkhatib A., Klonizakis M. (2015). Brief Report: Implementing a Mediterranean Diet Intervention into a RCT: Lessons Learned from a Non-Mediterranean Based Country. J. Nutr. Health Aging.

[100] World Health Organization Regional Office for the Western Pacific Regional Action Framework on Protecting Children from the Harmful Impact of Food Marketing in the Western Pacific. http://iris.wpro.who.int/handle/10665.1/14501.

[101] Onishi A., Furukawa T.A. (2014). Publication bias is underreported in systematic reviews published in high-impact-factor journals: Metaepidemiologic study. J. Clin. Epidemiol..

