# Supplementary material for: Measures of Perceived Neighborhood Food Environments and Dietary Habits: A Systematic Review of Methods and Associations
Source: Nutrients. 2022 Apr 24;14(9):1788. doi: 10.3390/nu14091788 (PMC9099956; doi:10.3390/nu14091788)
Supplement: Supplementary file 1 [file nutrients-14-01788-s001.zip › nutrients-1654171-supplementary.pdf]

Supplementary Materials

Table S1. Keyword search conducted on Pub Med

# Topic	Keywords[a]	Number of references
1. Food access	(perceived[TW]) OR (perception[TW]) OR (subjective[TW]) OR (perceived affordability[TW]) OR (subjective affordability[TW]) OR (perceived acceptability[TW]) OR (subjective acceptability[TW]) OR (accessibility) OR (availability[TW]) OR (affordability[TW]) OR (acceptability[TW]) OR (accommodation[TW])	231,043
2. Food environments	(Neighborhood*[TW]) OR (neighbourhood*[TW]) OR (“food environment*”[TW]) OR (communit*[TW]) OR (local[TW]) OR (“built environment*”[TW]) OR (foodscape[TW])	194,857
3. Food sources	(“food desert*”[TW]) OR (“food outlet*”[TW]) OR (“food store*”[TW]) OR (grocer*[TW]) OR (supermarket*[TW]) OR (super market*[TW]) OR (convenience store*[TW]) OR (“fast food*”[TW]) OR (restaurant*[TW]) OR (carryout*[TW]) OR (takeaway*[TW]) OR (“food supply”[TW]) OR (“corner store*”[TW]) OR (shop[TW]) OR (mall[TW]) OR (drive-in[TW]) OR (“eating place”[TW]) OR (retail*[TW]) OR (“food retail”[TW]) OR (“food deliver*”[TW]) OR (“food swamp*”[TW]) OR (“local food”[TW]) OR (“Food Industr*”[TW]) OR ("Food Supply"[TW]) OR ("Food Service*"[TW]) OR (“online market*”[TW])	7,267
4. Dietary habits	(diet*[TW]) OR (fruit*[TW]) OR (vegetable*[TW]) OR (“healthy diet*”[TW]) OR (“healthy food”[TW]) OR (“healthy consum*”[TW]) OR (“healthy intake*”[TW]) OR (“healthy eating”[TW]) OR (nutrition*[TW]) OR (consum*[TW]) OR (intake*[TW]) OR (eat[TW])	157,458
5.	#2 OR #3	199,916
6.	#1 AND #5	32,520
7.	#6 AND #4	4,225
8. Non-target population and disabilities	(child*[TW]) OR (youth*[TW]) OR (adolescent*[TW]) OR (school*[TW]) OR (student*[TW]) OR (worksite[TW]) OR (workplace[TW]) OR (work*[TW]) OR (employee*[TW]) OR (child*[TW]) OR (youth*[TW]) OR (adolescent*[TW]) OR (school*[TW]) OR (universit*[TW]) OR (worksite[TW]) OR (workplace[TW]) OR (work*[TW]) OR (hospital[TW]) OR (pharmac*[TW]) OR (drug*[TW]) OR (patient*[TW]) OR (nurs*[TW]) OR (treatment[TW]) OR (Medica*[TW]) OR (therap*[TW]) OR (emergenc*[TW]) OR (refuge*[TW]) OR (homeless[TW]) OR (pregnant*[TW]) OR (matern*[TW]) OR (“mental health”[TW]) OR (depress*[TW]) OR (HIV[TW]) OR (AIDS[TW]) OR (hygiene[TW]) OR (cancer*[TW]) OR (alcohol*[TW]) OR (athlete*[TW]) OR (climate[TW]) OR (disabilit*[TI]) OR (wheelchair*[TW]) OR (cardiovascular[TI]) OR (cardiometabolic[TI]) OR (CVD[TI]) OR (kidney[TI]) OR (CKD[TI]) OR (Diabet*[TI]) OR (Oral[TI]) OR (dental[TI]) OR (Stroke[TI]) OR (“heart attack”[TI]) OR (dementia[TI]) OR (Neuro*[TI]) OR (brain[TI]) OR (smok*[TI]) OR (Tobacco*[TI]) OR (cigarette*[TI]) OR (water[TI]) OR (exercise*[TI]) OR (muscle[TI]) OR (traine*[TI]) OR (sequenc*[TI]) OR (RNA[TI]) OR (Hormon*[TI]) OR	2,041,868

	("Supplemental nutrition"[TI]) OR (message*[TI]) OR (guideline*[TI])	
9. Non-target Article type	(Meta-Analysis[PT]) OR ("systematic review"[PT]) OR (review[PT]) OR (Biography[PT]) OR ("Classical Article"[PT]) OR ("Clinical Conference"[PT]) OR (Comment[PT]) OR ("Consensus Development Conference"[PT]) OR (Dictionary[PT]) OR (Directory[PT]) OR ("Duplicate Publication"[PT]) OR (Editorial[PT]) OR (Festschrift[PT]) OR (Guideline[PT]) OR ("Historical Article"[PT]) OR ("Newspaper Article"[PT]) OR ("Retracted Publication"[PT]) OR ("Retraction of Publication"[PT]) OR ("Twin Study"[PT])	116,053
10. Non-target field place (Low-income countries)	(Afghanistan) OR ("Guinea-Bissau") OR ("Sierra Leone") OR (Benin) (Haiti) OR (Somalia) OR ("Burkina Faso") OR ("South Sudan") OR (Burundi) OR (Liberia) OR ("Syrian Arab Republic") OR ("Central African Republic") OR (Madagascar) OR (Tajikistan) OR (Chad) OR (Malawi) OR (Tanzania) OR (Congo) OR (Mali) OR (Togo) OR (Eritrea) OR (Mozambique) OR (Uganda) OR (Ethiopia) OR (Nepal) OR ("Yemen, Rep.") OR ("Gambia, The") OR (Niger) OR (Guinea) OR (Rwanda)	24,163
11.	#7 NOT #8	546
12.	#11 NOT #9	528
13.	#12 NOT #10	516

TW: Text Words, TI: Title Words, PT: Publication Type

[a] Before searching with keywords, the filtration tool was used to ensure text availability (full text), target (humans), language (English), age (adult: 19 years and older) and publication year (2010–2020). We also extracted studies targeting adults aged 18 years and older, after filtration, though the filtration of age was set at '19 years and older'

Table S2. Keyword search conducted on the Web of Science

# Topic	Keywords[a]	Number of references
1. Food access	TS = (perceive OR perception OR subjective OR perceived affordability OR subjective affordability OR perceived acceptability OR subjective acceptability OR accessibility OR availability OR affordability OR acceptability OR accommodation)	510,138
2. Food environments	TS = (Neighborhood* OR neighbourhood* OR “food environment*” OR communit* OR local OR “built environment*” OR foodscape)	1,004,028
3 Food sources	TS = (“food desert*” OR “food outlet*” OR “food store*” OR grocer* OR supermarket* OR super market* OR convenience store* OR “fast food*” OR restaurant* OR carryout* OR takeaway* OR “food supply” OR “corner store*” OR shop OR mall OR drive-in OR “eating place” OR retail* OR “food retail” OR “food deliver*” OR “food swamp*” OR “local food” OR “Food Industr*” OR "Food Supply" OR "Food Service*" OR “online market*”)	59,739
4. Dietary habits	TS = (diet* OR fruit* OR vegetable* OR “healthy diet*” OR “healthy food” OR “healthy consum*” OR “healthy intake*” OR “healthy eating” OR nutrition* OR consum* OR intake* OR eat)	960,017
5.	#2 OR #3	1,054,114
6.	#1 AND #5	80,439
7.	#6 AND #4	13,793
8. Non-target population	TS = (child* OR youth* OR adolescent* OR school* OR student* OR worksite OR workplace OR work* OR employee* OR child* OR youth* OR adolescent* OR school* OR universit* OR worksite OR workplace OR work* OR hospital OR pharmac* OR drug* OR patient* OR nurs* OR treatment OR Medica* OR therap* OR emergenc* OR refuge* OR homeless OR pregnant* OR matern* OR “mental health” OR depress* OR HIV OR AIDS OR hygiene OR cancer* OR alcohol* OR athlete* OR climate OR wheelchair*)	6,369,574
9. Non-target Disabilities	TI = (disabilit* OR cardiovascular OR cardiometabolic OR CVD OR kidney OR CKD OR Diabet* OR Oral OR dental OR Stroke OR “heart attack” OR dementia OR Neuro* OR brain OR smok* OR Tobacco* OR cigarette* OR water OR exercise* OR muscle OR traine* OR sequenc* OR RNA OR Hormon* OR “Supplemental nutrition” OR message* OR guideline*)	1,108,861
10. Non-target field places (Low-income countries)	CU = (Afghanistan OR “Guinea-Bissau” OR “Sierra Leone” OR Benin Haiti OR Somalia OR “Burkina Faso” OR “South Sudan” OR Burundi OR Liberia OR “Syrian Arab Republic” OR “Central African Republic” OR Madagascar OR Tajikistan OR Chad OR Malawi OR Tanzania OR Congo OR Mali OR Togo OR Eritrea OR Mozambique OR Uganda OR Ethiopia OR Nepal OR “Yemen, Rep.” OR “Gambia, The” OR Niger OR Guinea OR Rwanda)	58,887
11.	#7 NOT #8	6,436
12.	#11 NOT #9	6,013
13.	#12 NOT #10	5,878

Filtration for excluding document type	Proceedings Paper OR Book Chapter OR Retracted Publication	5,876
Filtration for including research field	Environmental Sciences OR Food Science Technology OR Nutrition Dietetics OR Public Environmental Occupational Health OR Health Policy Services OR Environmental Studies OR Social Sciences Biomedical OR Gerontology OR Behavioral Sciences OR Anthropology	2,410

TS: Topic; TI: title, CU: Country/Region

[a] Before searching with keywords, the filtration tool was used to ensure text availability (articles), language (English), and publication year (2010–2020).

Table S3. Assessment sheet of the risk of bias [a]

**Bias due to confounding**
1.1 Is there potential for confounding of the effect (or the association) of the exposure (food environments) in this study?<br><br>✓ If No (N) or Probably no (PN),<br>Skip all remaining questions (1.2 to 1.8) and go to "Bias due to confounding: Risk of bias judgement"<br><br>✓ If Yes (Y) or Probably yes (PY)
1.2 Do statistical methods either account for clustering or multi-level models if neighborhood-level data were used?<br><br>✓ Y or PY<br><br>✓ N or PN
1.3. If Y or PY to 1.1, answer this question to determine whether there is a need to assess time-varying confounding: Was the analysis based on splitting follow-up time according to the exposure (food environments) received?<br>※If the study is cross-sectional study, select "N or PN".<br><br>✓ If N or PN, skip 1.4 and answer questions 1.5 to 1.7, which relate to baseline confounding.<br><br>✓ If Y or PY, go to question 1.4.
1.4. If Y or PY to 1.3: Were the exposure (food environments) discontinuations or switches likely to be related to factors that are prognostic for the outcome (dietary habits)?<br><br>✓ If N or PN, answer questions 1.5 to 1.7, which relate to baseline confounding only. Do not answer 1.8 and 1.9, which relate to both baseline and time-varying confounding.<br><br>✓ If Y or PY, skip questions 1.5 to 1.7, and answer questions 1.8 and 1.9, which relate to both baseline confounding and time-varying confounding.
1.5. If N or PN to 1.3 or 1.4: Did the authors use an appropriate analysis method that adjusted for all the critically important confounding variables at baseline?<br><br>✓ If Y or PY, Go to 1.6
1.6. Were confounders that were adjusted for measured validly and reliably by the variables available in this study?<br><br>✓ If Y or PY, Go to 1.7
1.7. Did the authors avoid adjusting for variables (post-exposure variables in cohort study)?<br><br>(Skip to Bias due to confounding: Risk of bias judgement)
**Questions related to baseline and time-varying confounding**<br><br>1.8. If Y or PY to 1.4: Did the authors use an appropriate analysis method that adjusted for all the critically important confounding variables (i.e., age, race, sex, socioeconomic status), including baseline and time-varying confounding?<br><br>✓ If N or PN to 1.8, skip to "Bias due to confounding: Risk of bias judgement".<br><br>✓ If Y or PY to 1.8, answer question 1.9.
1.9. If Y or PY to 1.4 and Y or PY to 1.8: Were confounders that were adjusted for measured validly

and reliably by the variables available in this study?<br><br>✓ Y or PY<br><br>✓ N or PN

**Bias due to confounding: Risk of bias judgement; the study can be considered to be at low risk of bias due to confounding and no further signaling questions need be considered.**	
Low risk of bias (the study is comparable to a well-performed randomized trial with regard to this domain)	No confounding expected.
Moderate risk of bias (the study is sound for an observational study with regard to this domain but cannot be considered comparable to a well-performed randomized trial)	(i) Confounding expected, all known important confounding domains appropriately measured and controlled for;<br>and<br>(ii) Reliability and validity of measurement of important domains were sufficient, such that we do not expect serious residual confounding.
Serious risk of bias (the study has some important problems)	(i) At least one key confounder was not appropriately measured, or not controlled for;<br>or<br>(ii) Reliability or validity of measurement of a key confounder was low enough that we expect serious residual confounding.
Critical risk of bias (the study is too problematic to provide any useful evidence on the effects [or the associations] of exposure)	(i) Confounding is inherently not controllable;<br>or<br>(ii) The use of negative controls strongly suggests unmeasured confounding.
No information on which to base a judgement about risk of bias for this domain	No information on whether confounding might be present

**Bias in selection of participants into the study**
2.1. Was selection of participants into the study or into the analysis based on participant characteristics observed after the start of (or simultaneously in the cross-sectional study) the exposure (food environments)?<br><br>✓ If N or PN, go to 2.4 (skip 2.2 and 2.3).<br><br>✓ If Y or PY, go to 2.2 and 2.3.
2.2. If Y or PY to 2.1: Were the post-exposure variables that influenced selection of participants (into the study or analysis) associated with the exposure (food environments)?<br><br>✓ If Y or PY, Go to 2.3
2.3. If Y or PY to 2.1: Were the post-exposure variables that influenced selection of participants (into the study or analysis) associated with the outcome (dietary habits)?<br><br>✓ If Y or PY, Go to 2.4
2.4. Do start of follow-up and start of the exposure (food environments) coincide for most participants?<br><br>✓ If N or PN to 2.4, answer 2.5.

✓ If Y or PY to 2.4, go to Bias in selection of participants into the study: Risk of bias judgement.	
2.5 If Y or PY to 2.2 and 2.3, or N or PN to 2.4: Were adjustment techniques that were likely to correct for the presence of selection biases used?<br><br>✓ Go to "Bias in selection of participants into the study: Risk of bias judgement".	
**Bias in selection of participants into the study: Risk of bias judgement**	
Low risk of bias (the study is comparable to a well-performed randomized trial with regard to this domain)	(i) All participants who would have been eligible for the target trial were included in the study;<br>and<br>(ii) For each participant, start of follow up and start of the exposure (food environments) coincided.
Moderate risk of bias (the study is sound for an observational study with regard to this domain but cannot be considered comparable to a well-performed randomized trial)	(i) Selection into the study may have been related to the exposure (food environments) and the outcome (dietary habits);<br>and<br>The authors used appropriate methods to adjust for the selection bias;<br>or<br>(ii) Start of follow up and start of the exposure (food environments) do not coincide for all participants;<br>and<br>(a) the proportion of participants for which this was the case was too low to induce important bias;<br>or<br>(b) the authors used appropriate methods to adjust for the selection bias;<br>or<br>(c) the review authors are confident that the rate (hazard) ratio for the effect (or the association) of the exposure (food environments) remains constant over time.
Serious risk of bias (the study has some important problems)	(i) Selection into the study was related (but not very strongly) to the exposure (food environments) and the outcome (dietary habits);<br>and<br>This could not be adjusted for in analyses;<br>or<br>(ii) Start of follow up and start of the exposure (food environments) do not coincide;<br>and<br>A potentially important amount of follow-up time is missing from analyses;<br>and<br>The rate ratio is not constant over time.
Critical risk of bias (the study is too problematic to provide any useful evidence on the effects [or the associations] of the exposure)	(i) Selection into the study was very strongly related to the exposure (food environments) and the outcome (dietary habits);<br>and<br>This could not be adjusted for in analyses;<br>Or<br>(ii) A substantial amount of follow-up time is likely to be missing from analyses;<br>and

	The rate ratio is not constant over time.
No information on which to base a judgement about risk of bias for this domain	No information is reported about selection of participants into the study or whether start of follow up and start of the exposure (food environments) coincide.

**Bias in classification of exposures**
3.1. Is the exposure (food environments) that was assessed clearly defined?<br><br>✓ Y or PY<br><br>✓ N or PN
3.2. Does the exposure (food environments) that was assessed represent the exposure of interest?<br><br>✓ Y or PY<br><br>✓ N or PN
3.3. Were the methods used to assess the exposure (food environments) clearly described?<br><br>✓ Y or PY<br><br>✓ N or PN
3.4. Were the methods used to measure the exposure (food environments) valid and/or reliable?<br><Need to concern><br>a) Exposure assignment based on a neighborhood or administrative unit rather than individual location (or block). (The individual location is more precise measurement)<br>b) Food outlet data not validated in person.<br>※ Establishing the convergent validity of the proposed measure against alternative measures of the environmental dimension, recognizing that there is currently no accepted "gold standard"[4].<br><br>✓ Y or PY<br><br>✓ N or PN
3.5. Were the same methods used to assess the exposure (food environments) status for all participants/groups?<br><br>✓ Y or PY<br><br>✓ N or PN
3.6. Were the methods used to define the exposure (food environments) status for participants/groups clearly described?<br><br>✓ Y or PY<br><br>✓ N or PN
3.8. Could classification of the exposure (food environments) status been affected by the presence of the outcome (dietary habits), knowledge of the outcome (dietary habits) or risk of the outcome (dietary habits)?<br><br>✓ If Y or PY, there may be serious risk of bias.<br><br>Go to Bias in classification of the exposure (food environments): Risk of bias judgement.

**Bias in classification of exposures: Risk of bias judgement**	
Low risk of bias (the study is comparable to a well-performed randomized trial with regard to this domain)	(i) The exposure (food environments) and the methods used to assess the exposure (food environments) were well defined and represent the exposure (food environments) of interest;<br>and<br>(ii) Methods were valid, reliable, the same across groups.<br>and<br>(iii) Exposure (food environments) status was not affected by the presence of the outcome (dietary habits), knowledge of the outcome (dietary habits) or risk of the outcome (dietary habits)
Moderate risk of bias (the study is sound for a, observational study with regard to this domain but cannot be considered comparable to a well-performed randomized trial)	(i) The exposure (food environments) and the methods used to assess the exposure (food environments) are defined and represent the exposure (food environments) of interest;<br>and<br>(ii) Methods were valid, reliable, the same across groups.<br>or<br>Exposure (food environments) status was not affected by the presence of the outcome (dietary habits), knowledge of the outcome (dietary habits) or risk of the outcome (dietary habits)
Serious risk of bias (the study has some important problems)	(i) Exposure (food environments) status or the methods used to assess the exposure (food environments) are not well defined or do not represent the exposure of interest;<br>and<br>(ii) Methods were not valid and reliable, the same across groups.<br>or<br>Exposure (food environments) status was affected by the presence of the outcome (dietary habits), knowledge of the outcome (dietary habits) or risk of the outcome (dietary habits)
Critical risk of bias (the study is too problematic to provide any useful evidence on the effects [or the association] of exposure)	(i) Exposure (food environments) status and the methods used to assess the exposure (food environments) are not well defined or do not represent the exposure (food environments) of interest;<br>and<br>(ii) Methods were not valid and reliable, were not the same across groups.<br>And<br>(iii) Exposure (food environments) status was affected by the presence of the outcome (dietary habits), knowledge of the outcome (dietary habits) or risk of the outcome (dietary habits)
No information on which to base a judgement about risk of bias for this domain	No definition of the exposure (food environments) or no explanation of the source of information about the exposure (food environments) status is reported.

**Bias due to departures from intended exposures**
4.1. Is there concern that changes in the exposure (food environments) status occurred among participants that

were unbalanced across groups and likely to impact the outcome (dietary habits)?<br><br>✓ Y or PY<br><br>✓ N or PN
4.2. Were any critical co-exposures that occurred unbalanced between exposure (food environments) groups and likely to impact the outcome (dietary habits)?<br><br>✓ Y or PY<br><br>✓ N or PN, go to Bias due to departures from intended exposures: Risk of bias judgement (skip 4.3).
4.3. If Y or PY to 4.1, or 4.2: Were adjustment techniques that are likely to correct for these issues (i.e., changes in the exposure (food environments) status and/or unbalanced co-exposures used?<br><br>✓ Y or PY<br><br>✓ N or PN<br><br>Go to Bias due to departures from intended exposures: Risk of bias judgement.

**Bias due to departures from intended exposures: Risk of bias judgement**	
Low risk of bias (the study is comparable to a well-performed randomized trial with regard to this domain)	There were no changes in the exposure (food environments) status that were likely to impact the outcome (dietary habits), and any important co-exposures were balanced across exposure groups.
Moderate risk of bias (the study is sound for an observational study with regard to this domain but cannot be considered comparable to a well-performed randomized trial)	(i) There were changes in the exposure (food environments) status or important co-exposures were not balanced across groups<br>and<br>(ii) The impact on the outcome (dietary habits) is expected to be slight or<br>measurement and/or adjustment techniques were used to correct for the issues
Serious risk of bias (the study has some important problems)	(i) There were changes in the exposure (food environments) status or important co-exposures were not balanced across groups that were likely to impact the outcome (dietary habits), and<br>(ii) No or inappropriate measurement and/or adjustment techniques were used to correct for the issues
Critical risk of bias (the study is too problematic to provide any useful evidence on the effects [or the associations] of the exposure)	(i) There were substantial changes in the exposure (food environments) status, or important co-exposure were not balanced across groups, that were likely to impact the outcome (dietary habits),<br>and<br>(ii) No or inappropriate measurement and/or adjustment techniques were used to correct for the issues.
No information on which to base a judgement about risk of bias for this domain	No information is reported on whether there is deviation from the intended exposure (food environments).

**Bias due to missing data**
5.1. Were there missing outcome (dietary habits) data?

<table>
<tr><td colspan="2">✓ Y or PY

✓ N or PN</td></tr>
<tr><td colspan="2">5.2. Were participants excluded due to missing data on the exposure (food environments) status?

✓ Y or PY

✓ N or PN</td></tr>
<tr><td colspan="2">5.3. Were participants excluded due to missing data on other variables (besides outcome (dietary habits) data and the exposure (food environments) status) needed for the analysis?

✓ Y or PY

✓ N or PN</td></tr>
<tr><td colspan="2">5.4. If Y or PY to 5.1, 5.2 or 5.3: Are the proportion of participants and reasons for missing data similar across the exposure (food environments) groups?

✓ Y or PY

✓ N or PN</td></tr>
<tr><td colspan="2">5.5. If Y or PY to 5.1, 5.2 or 5.3: Were appropriate statistical methods used to account for missing data?

✓ Y or PY

✓ N or PN

Go to Bias due to missing data: Risk of bias judgement</td></tr>
<tr><td colspan="2">Bias due to missing data: Risk of bias judgement</td></tr>
<tr><td>Low risk of bias (the study is comparable to a well-performed randomized trial with regard to this domain)</td><td>(i) Data were reasonably complete;
or
(ii) Proportions of and reasons for missing participants were similar across the exposure (food environments) groups;
or
(iii) The analysis addressed missing data and is likely to have removed any risk of bias.</td></tr>
<tr><td>Moderate risk of bias (the study is sound for an observational study with regard to this domain but cannot be considered comparable to a well-performed randomized trial)</td><td>(i) Proportions of and reasons for missing participants differ slightly across the exposure (food environments) groups;
and
(ii) The analysis is unlikely to have removed the risk of bias arising from the missing data.</td></tr>
<tr><td>Serious risk of bias (the study has some important problems)</td><td>(i) Proportions of missing participants differ substantially across the exposure (food environments);
or
Reasons for missingness differ substantially across the exposure (food environments);
and
(ii) The analysis is unlikely to have removed the risk of bias arising from the missing data;
or
Missing data were addressed inappropriately in the analysis;
or
The nature of the missing data means that the risk of bias cannot be removed through appropriate analysis.</td></tr>
<tr><td>Critical risk of bias (the study is too</td><td>(i) (Unusual) There were critical differences between the</td></tr>
</table>

problematic to provide any useful evidence on the effects [or the associations] of the exposure)	exposure (food environments) in participants with missing data;<br>and<br>(ii) Missing data were not, or could not, be addressed through appropriate analysis.
No information on which to base a judgement about risk of bias for this domain	No information is reported about missing data or the potential for data to be missing.

**Bias in measurement of outcomes**	
6.1. Could the outcome (dietary habits) measure have been influenced by knowledge of the exposure (food environments) received?<br><br>✓ Y or PY<br><br>✓ N or PN	
6.2. Were outcome (dietary habits) assessors aware of the exposure (food environments) received by study participants?<br><br>✓ Y or PY<br><br>✓ N or PN	
6.3. Were the methods of outcome (dietary habits) assessment the same across the exposure (food environments) groups?<br><br>✓ Y or PY<br><br>✓ N or PN	
6.4. Were dietary habits reflecting the intake of fruits and vegetables measured by a dietary record (i.e., weighed food record) maintained by trained staff?<br><br>✓ If Y or PY, skip to 6.5 and go to 6.6.<br><br>✓ If N or PN, answer question 6.5.	
6.5. The self-reported food frequency questionnaire, 24-hour dietary recall, diet quality score, frequency of fast food intake, and other measurements for dietary habits were validated.<br><br>✓ Y or PY<br><br>✓ N or PN	
6.6. Did any systematic errors emerge during the measurement of the outcome (dietary habits) related to the exposure (food environments)?<br><br>✓ Y or PY<br><br>✓ N or PN<br><br>Go to Bias in measurement of outcomes (dietary habits): Risk of bias judgement	
**Bias in measurement of outcomes: Risk of bias judgement**	
Low risk of bias (the study is comparable to a well-performed randomized trial with regard to this domain)	(i) The methods of outcome (dietary habits) assessment were comparable across the exposure (food environments) groups;<br>and

	(ii) The outcome (dietary habits) measure was unlikely to be influenced by knowledge of the exposure (food environments) received by study participants (i.e. is objective) or the outcome (dietary habits) assessors were unaware of the exposure (food environments) received by study participants;<br>and<br>(iii) Any error in measuring the outcome (dietary habits) is unrelated to the exposure (food environments) status.<br>and<br>(iv) The dietary habits were documented in the dietary record (i.e., weighed food record) maintained by trained staffs or self-reported measure confirming the validation.
Moderate risk of bias (the study is sound for an observational study with regard to this domain but cannot be considered comparable to a well-performed randomized trial)	(i) The methods of outcome (dietary habits) assessment were comparable across the exposure (food environments) groups;<br>and<br>(ii) The outcome (dietary habits) measure is only minimally influenced by knowledge of the exposure (food environments) received by study participants;<br>and<br>(iii) Any error in measuring the outcome (dietary habits) is only minimally related to the exposure (food environments) status.<br>and<br>(iv) The measurements of dietary habits were self-reported. They were not validated but comparatively reliable.
Serious risk of bias (the study has some important problems)	(i) The methods of outcome (dietary habits) assessment were not comparable across the exposure (food environments) groups;<br>or<br>(ii) The outcome (dietary habits) measure was subjective (i.e. vulnerable to influence by knowledge of the exposure (food environments) received by study participants);<br>and<br>The outcome (dietary habits) was assessed by assessors aware of the exposure (food environments) received by study participants;<br>or<br>(iii) Error in measuring the outcome (dietary habits) was related to the exposure (food environments) status.<br>and<br>(iv) The measurements of dietary habits were self-reported, not validated and not reliable.
Critical risk of bias (the study is too problematic to provide any useful evidence on the effects [or the associations] of exposure)	The methods of outcome (dietary habits) assessment were so different that they cannot reasonably be compared across the exposure groups.
No information on which to base a judgement about risk of bias for this domain	No information is reported about the methods of outcome (dietary habits) assessment.

**Bias in selection of reported result**
7.1. Is the reported effect (or the association) estimate likely to be selected on the basis of the results from multiple outcome (dietary habits) measurements within the outcome domain?

✓ Y or PY<br><br>✓ N or PN	
7.2. Is the reported effect (or the association) estimate likely to be selected on the basis of the results from multiple analyses of the exposure (food environments)-outcome (dietary habits) relationship?<br><br>✓ Y or PY<br><br>✓ N or PN	
7.3. Is the reported effect (or the association) estimate likely to be selected on the basis of the results from different subgroups?<br><br>✓ Y or PY<br><br>✓ N or PN<br><br>Go to Bias in selection of reported result: Risk of bias judgement	
**Bias in selection of reported result: Risk of bias judgement**	
Low risk of bias (the study is comparable to a well-performed randomized trial with regard to this domain)	There is clear evidence (usually through examination of a pre-registered protocol or statistical analysis plan) that all reported results correspond to all intended outcomes (dietary habits), analyses and sub-cohorts.
Moderate risk of bias (the study is sound for an observational study with regard to this domain but cannot be considered comparable to a well-performed randomized trial)	(i) The outcome (dietary habits) measurements and analyses are consistent with an a priori plan; or are clearly defined and both internally and externally consistent;<br>and<br>(ii) There is no indication of selection of the reported analysis from among multiple analyses;<br>and<br>(iii) There is no indication of selection of the cohort or subgroups for analysis and reporting on the basis of the results.
Serious risk of bias (the study has some important problems)	(i) Outcomes (dietary habits) are defined in different ways in the methods and results sections, or in different publications of the study;<br>or<br>(ii) There is a high risk of selective reporting from among multiple analyses;<br>or<br>(iii) The cohort or subgroup is selected from a larger study for analysis and appears to be reported on the basis of the results.
Critical risk of bias (the study is too problematic to provide any useful evidence on the effects [or the associations] of the exposure)	(i) There is evidence or strong suspicion of selective reporting of results;<br>and<br>(ii) The unreported results are likely to be substantially different from the reported results.
No information on which to base a judgement about risk of bias for this domain	There is too little information to make a judgement (for example if only an abstract is available for the study).

[a] The items of the risk of bias were listed according to the previous reports (Bero et al., 2018; Hörnell et al., 2017; Wells GA et al. [accessed March 9, 2022]).

Table S4. Results of the risk of bias assessment [a]

Reference	Bias due to confounding	Bias in selection of participants into the study	Bias in classification of exposures	Bias due to departures from intended exposures	Bias due to missing data	Bias in measurement of outcomes	Bias in selection of reported results
Alber et al., 2018	Moderate	Moderate	Low	Moderate	NI	Moderate	Moderate
Bivoltsis et al., 2020	Moderate	Moderate	Low	Moderate	Moderate	Moderate	Moderate
Carbonneau et al., 2019	Moderate	Moderate	Low	Moderate	Low	Moderate	Moderate
Caspi et al., 2012	Moderate	Moderate	Low	Moderate	NI	Low	Moderate
Chapman et al., 2017	Moderate	Moderate	Low	Moderate	Moderate	Moderate	Moderate
Flint et al., 2013	Moderate	Moderate	Low	Moderate	Moderate	Low	Moderate
Freedman et al., 2019	Moderate	Moderate	Low	Moderate	Low	Low	Moderate
Gase et al., 2016	Moderate	Moderate	Low	Moderate	Moderate	Moderate	Moderate
Jilcott Pitts et al., 2015	Moderate	Moderate	Low	Moderate	NI	Moderate	Moderate
Kegler et al., 2014	Moderate	Moderate	Low	Moderate	NI	Moderate	Moderate
Liese et al., 2014	Moderate	Moderate	Low	Moderate	Moderate	Low	Moderate
Lo et al., 2019	Moderate	Moderate	Low	Moderate	Low	Moderate	Moderate
Lucan and Mitra, 2012	Moderate	Moderate	Low	Moderate	Low	Low	Moderate
Ma et al., 2018	Moderate	Moderate	Low	Moderate	Moderate	Low	Moderate
Minaker et al., 2013	Moderate	Moderate	Low	Moderate	NI	Moderate	Moderate
Oexle et al., 2015	Moderate	Moderate	Low	Moderate	Moderate	Low	Moderate
Sharkey et al., 2010	Moderate	Moderate	Moderate	Moderate	Moderate	Moderate	Moderate
Springvloet et al., 2014	Moderate	Moderate	Moderate	Moderate	Moderate	Moderate	Moderate
Yamaguchi et al., 2019	Moderate	Moderate	Moderate	Moderate	Moderate	Moderate	Moderate

Abbreviation – NI: no information

[a] The risk of bias in each domain was rated on a five-point scale: low, moderate, serious, critical, and no information.

References

Alber, J.M.; Green, S.H.; Glanz, K. Perceived and Observed Food Environments, Eating Behaviors, and BMI. *Am J Prev Med* **2018**, *54*, 423-429, doi:10.1016/j.amepre.2017.10.024.

Bero, L.; Chartres, N.; Diong, J.; Fabbri, A.; Ghersi, D.; Lam, J.; Lau, A.; McDonald, S.; Mintzes, B.; Sutton, P., et al. The risk of bias in observational studies of exposures (ROBINS-E) tool: concerns arising from application to observational studies of exposures. *Systematic reviews* **2018**, *7*, 242, doi:10.1186/s13643-018-0915-2.

Bivoltsis, A.; Trapp, G.; Knuiman, M.; Hooper, P.; Ambrosini, G.L. The influence of the local food environment on diet following residential relocation: longitudinal results from RESIDential Environments (RESIDE). *Public Health Nutr* **2020**, *23*, 2132-2144, doi:10.1017/S1368980019005111.

Carbonneau, E.; Lamarche, B.; Robitaille, J.; Provencher, V.; Desroches, S.; Vohl, M.C.; Begin, C.; Belanger, M.; Couillard, C.; Pelletier, L., et al. Social Support, but Not Perceived Food Environment, Is Associated with Diet Quality in French-Speaking Canadians from the PREDISE Study. *Nutrients* **2019**, *11*, 3030, doi:10.3390/nu11123030.

Caspi, C.E.; Kawachi, I.; Subramanian, S.V.; Adamkiewicz, G.; Sorensen, G. The relationship between diet and perceived and objective access to supermarkets among low-income housing residents. *Social Science & Medicine* **2012**, *75*, 1254-1262, doi:10.1016/j.socscimed.2012.05.014.

Chapman, K.; Goldsbury, D.; Watson, W.; Havill, M.; Wellard, L.; Hughes, C.; Bauman, A.; Allman-Farinelli, M. Exploring perceptions and beliefs about the cost of fruit and vegetables and whether they are barriers to higher consumption. *Appetite* **2017**, *113*, 310-319, doi:10.1016/j.appet.2017.02.043.

Flint, E.; Cummins, S.; Matthews, S. Do perceptions of the neighbourhood food environment predict fruit and vegetable intake in low-income neighbourhoods? *Health & Place* **2013**, *24*, 11-15, doi:10.1016/j.healthplace.2013.07.005.

Freedman, D.A.; Bell, B.A.; Clark, J.K.; Sharpe, P.A.; Trapl, E.S.; Borawski, E.A.; Pike, S.N.; Rouse, C.; Sehgal, A.R. Socioecological Path Analytic Model of Diet Quality among Residents in Two Urban Food Deserts. *J Acad Nutr Diet* **2019**, *119*, 1150-1159, doi:10.1016/j.jand.2019.02.012.

Garriguet, D. Diet quality in Canada. *Health Rep* **2009**, *20*, 41-52.

Gase, L.N.; Glenn, B.; Kuo, T. Self-Efficacy as a Mediator of the Relationship Between the Perceived Food Environment and Healthy Eating in a Low Income Population in Los Angeles County. *J Immigr Minor Health* **2016**, *18*, 345-352, doi:10.1007/s10903-015-0186-0.

Hörnell, A.; Berg, C.; Forsum, E.; Larsson, C.; Sonestedt, E.; Åkesson, A.; Lachat, C.; Hawwash, D.; Kolsteren, P.; Byrnes, G., et al. Perspective: An Extension of the STROBE Statement for Observational Studies in Nutritional Epidemiology (STROBE-nut): Explanation and Elaboration. *Adv Nutr (Bethesda, Md.)* **2017**, *8*, 652-678, doi:10.3945/an.117.015941.

Jilcott Pitts, S.B.; Keyserling, T.C.; Johnston, L.F.; Smith, T.W.; McGuirt, J.T.; Evenson, K.R.; Rafferty, A.P.; Gizlice, Z.; Garcia, B.A.; Ammerman, A.S. Associations between neighborhood-level factors related to a healthful lifestyle and dietary intake, physical activity, and support for obesity prevention polices among rural adults. *J Community Health* **2015**, *40*, 276-284, doi:10.1007/s10900-014-9927-6.

Liese, A.D.; Bell, B.A.; Barnes, T.L.; Colabianchi, N.; Hibbert, J.D.; Blake, C.E.; Freedman, D.A. Environmental influences on fruit and vegetable intake: results from a path analytic model. *Public Health Nutr* **2014**, *17*, 2595-2604, doi:10.1017/S1368980013002930.
Lo, B.K.; Loui, C.; Folta, S.C.; Flickinger, A.; Connor, L.M.; Liu, E.; Megiel, S.; Seguin, R.A. Self-efficacy and cooking confidence are associated with fruit and vegetable intake in a cross-sectional study with rural women. *Eat Behav* **2019**, *33*, 34-39, doi:10.1016/j.eatbeh.2019.02.005.
Lucan, S.C.; Mitra, N. Perceptions of the food environment are associated with fast-food (not fruit-and-vegetable) consumption: findings from multi-level models. *Int J Public Health* **2012**, *57*, 599-608, doi:10.1007/s00038-011-0276-2.
Ma, X.N.; Blake, C.E.; Barnes, T.L.; Bell, B.A.; Liese, A.D. What does a person's eating identity add to environmental influences on fruit and vegetable intake? *Appetite* **2018**, *120*, 130-135, doi:10.1016/j.appet.2017.08.025.
Minaker, L.M.; Raine, K.D.; Wild, T.C.; Nykiforuk, C.I.; Thompson, M.E.; Frank, L.D. Objective food environments and health outcomes. *Am J Prev Med* **2013**, *45*, 289-296, doi:10.1016/j.amepre.2013.05.008.
Oexle, N.; Barnes, T.L.; Blake, C.E.; Bell, B.A.; Liese, A.D. Neighborhood fast food availability and fast food consumption. *Appetite* **2015**, *92*, 227-232, doi:10.1016/j.appet.2015.05.030.
Sharkey, J.R.; Johnson, C.M.; Dean, W.R. Food Access and Perceptions of the Community and Household Food Environment as Correlates of Fruit and Vegetable Intake among Rural Seniors. *BMC geriatrics* **2010**, *10*, doi:10.1186/1471-2318-10-32.
Springvloet, L.; Lechner, L.; Oenema, A. Can individual cognitions, self-regulation and environmental variables explain educational differences in vegetable consumption?: a cross-sectional study among Dutch adults. *Int J Behav Nutr Phys Act* **2014**, *11*, 149.
Wells GA; Shea B; O'Connell D; Peterson J; Welch V; Losos M; Tugwell P. The Newcastle-Ottawa Scale (NOS) for assessing the quality of nonrandomised studies in meta-analyses. Availabe online: http://www.ohri.ca/programs/clinical_epidemiology/oxford.asp (accessed on 9 March 2022).
Yamaguchi, M.; Takahashi, K.; Hanazato, M.; Suzuki, N.; Kondo, K.; Kondo, N. Comparison of Objective and Perceived Access to Food Stores Associated with Intake Frequencies of Vegetables/Fruits and Meat/Fish among Community-Dwelling Older Japanese. *Int J Environ Res Public Health* **2019**, *16*, doi:10.3390/ijerph16050772.
